# [{SiN^Dipp^}MgNa]_2_: A Potent Molecular Reducing Agent

**DOI:** 10.1021/acs.organomet.4c00076

**Published:** 2024-04-09

**Authors:** Han-Ying Liu, Samuel E. Neale, Michael S. Hill, Mary F. Mahon, Claire L. McMullin, Emma Richards

**Affiliations:** † Department of Chemistry, University of Bath, Claverton Down, Bath BA2 7AY, U.K.; ‡ School of Chemistry, Cardiff University, Main Building, Park Place, Cardiff CF10 3AT, U.K.

## Abstract

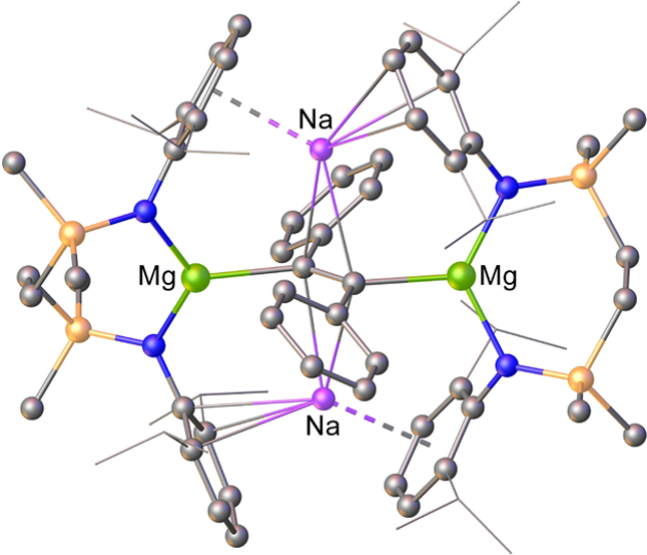

The bimetallic species, [{SiN^Dipp^}MgNa]_2_ [{SiN^Dipp^} = {CH_2_SiMe_2_N(Dipp)}_2_; (Dipp = 2,6-*i*-Pr_2_C_6_H_3_)], is shown to be a potent reducing agent, able to effect one- or two-electron reduction of either dioxygen, TEMPO, anthracene, benzophenone, or diphenylacetylene. In most cases, the bimetallic reaction products imply that the dissimilar alkaline metal centers react with a level of cooperativity. EPR analysis of the benzophenone-derived reaction and the concurrent isolation of [{SiN^Dipp^}Mg(OCPh_2_)_2_], however, illustrate that treatment with such reducible, but *O*-basic, species can also result in reactivity in which the metals provide independent reaction products. The notable *E*-stereochemistry of the diphenylacetylene reduction product prompted a computational investigation of the PhC≡CPh addition. This analysis invokes a series of elementary steps that necessitate ring-opening via Mg^+^ → Na^+^ amido group migration of the SiN^Dipp^ ligand, providing insight into the previously observed lability of the bidentate dianion and its consequent proclivity toward macrocyclization.

## Introduction

**Figure 1:**
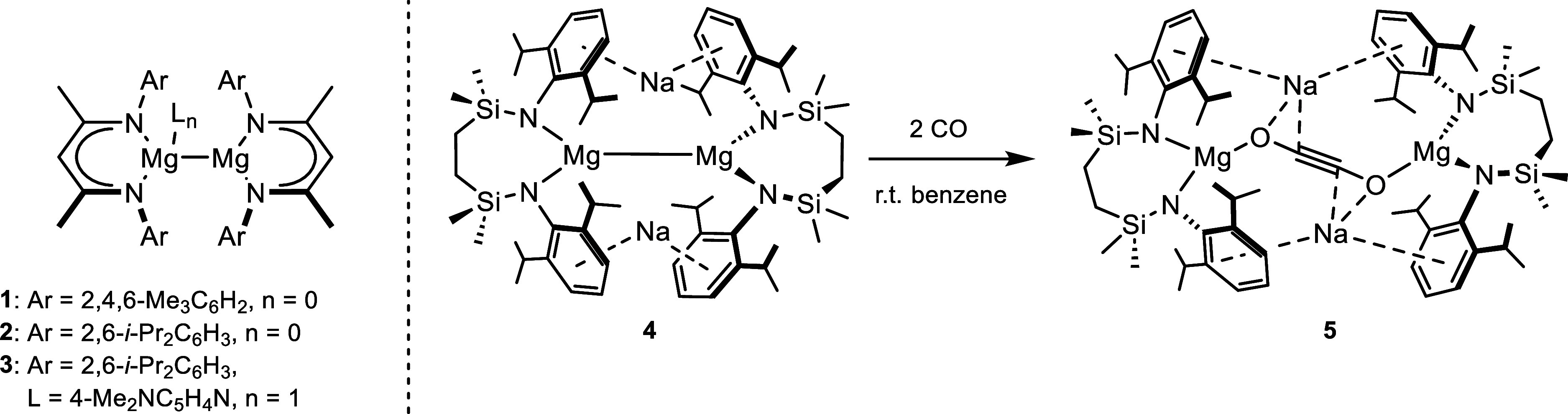
Structures of compounds **1**–**3** and the transformation of compound **4** to compound **5**.

Jones and co-workers’ 2007 demonstration of dimeric Mg(I) molecules (e.g., **1** and **2**, Figure 1) proved a landmark in main group chemistry and initiated a flourishing subfield in low oxidation state group 2 element synthesis.^1−4^ During the subsequent decade and a half, more than 30 further examples of compounds comprising unsupported Mg–Mg σ bonds have been described,^5−17^ while, with varying degrees of success, more recent endeavors have sought to access analogous or closely related species derived from magnesium’s lighter and heavier group 2 congeners.^4,18−23^

Theoretical and experimental charge density analysis of the intermetallic bonding (*r*_Mg–Mg_ = ca. 2.8–2.9 Å) of molecules exemplified by **1** and **2** (Figure 1) indicates a local maximum in the electron density between the magnesium atoms and the characterization of a so-called non-nuclear attractor.^24−26^ Although this latter feature exerts only limited structural consequences, the reactivity of such species has, at least in part, been attributed to the resultant and relatively weak association of the highest energy bonding electrons to each magnesium. Compounds **1** and **2**, therefore, have been shown to present a broad palette of reactivity as 2 electron reductants toward both organic and metallo-organic small-molecule substrates.^27^ While such species have, thus far, proved resistant to any reliable electrochemical estimate of their reduction potentials (*E*^0^), their ability to reduce, for example, anthracene (*E*^0^ = −1.98 V versus SCE) to its dianion and the isolation of a sixfold fullerene reduction product places them as at least competitive with the most potent chemical reductants.^28,29^

Despite the thermodynamic viability of the reactions,^30^ neither **1** nor **2** react directly with the archetypal small molecule, H_2_. Similarly, no reaction is observed when **1** is treated with CO.^8^ Its desymmetrization with a single equivalent of 4-dimethylaminopyridine, however, provides compound **3** (Figure 1), in which the reactivity of the elongated Mg–Mg bond (*r*_Mg–Mg_ = 3.089(1) Å) is sufficiently enhanced to enable reductive CO trimerization to a planar, aromatic deltate dianion, [C_3_O_3_]^2–^.^14^ In more recent extensions to this oligomerization chemistry, the Jones group has shown that the introduction of Mo(CO)_6_ into this and related Mg(I) systems provides higher oligomeric squarate, [C_4_O_4_]^2–^,^31^ and even benzenehexolate, [C_6_O_6_]^6–^ anion formation.^32^

In related chemistry, we have reported the synthesis of [{SiN^Dipp^}MgNa]_2_ (**4**, Figure 1; [{SiN^Dipp^} = {CH_2_SiMe_2_N(Dipp)}_2_; Dipp = 2,6-*i*-Pr_2_C_6_H_3_)]. Formal oxidation states of Na(I) and Mg(I) may be attributed to the dissimilar metal centers of compound **4**, which reacts with CO to provide the ethynediolate derivative (**5**, Figure 1).^33^ Compound **4** also reacts directly with H_2_, albeit this latter transformation is complicated by apparent disproportionation of 50% of the constituent magnesium in the reaction.^34^ Initial quantum theory of atoms in molecules (QTAIM) analysis of **4** led us to suggest that this reactivity is abetted by the contiguous {Na_2_Mg_2_} ensemble, which gives rise to an unusual manifold of frontier molecular orbitals wherein the closely separated (ca. 3 eV) HOMO and LUMO are largely derived from the 3s valence atomic wave functions of the magnesium and sodium atoms, respectively. An inference of intermetallic communication is underscored by the treatment of **4** with nonreducible bases such as THF. In such cases, the molecule is prone to complete extrusion of the Na^+^ component as metallic sodium in a process of self-reduction and Mg(I) oxidation, albeit the chelated {SiN^Dipp^} ligand is also observed to display a significant degree of lability resulting in the formation of a bimetallic macrocycle in which two diamides bridge between two now Mg(II) centers.^35^

This latter observation raises a pertinent question as to whether the reductive reactivity of **4** that provides compound **5** and the outcome of its interaction with H_2_ are truly cooperative processes or better characterized as an outcome of the *in situ* generation of atomic sodium. Attempting to shed further light on this issue, we report a study of the reactivity of compound **4** with a variety of representative molecular oxidants.

## Experimental Section

### General Considerations

Unless stated otherwise, all of the experiments were conducted using standard Schlenk line and/or glovebox techniques under an inert atmosphere of argon. NMR spectra were recorded with an Agilent ProPulse spectrometer (^1^H at 500 MHz, ^13^C at 126 MHz). The spectra are referenced relative to residual protio solvent resonances. Elemental analyses were performed at Elemental Microanalysis Ltd., Okehampton, Devon, UK. Solvents were dried by passage through a commercially available solvent purification system and stored under argon in ampules over 4 Å molecular sieves. C_6_D_6_ was purchased from Sigma-Aldrich and dried over a potassium mirror before distilling and storage over molecular sieves. {CH_2_SiMe_2_N(H)Dipp}_2_ and [{SiN^Dipp^}MgNa]_2_ (**4**) were synthesized according to literature procedures.^33a,36^ Benzophenone and TEMPO were purchased from Merck and purified by sublimation prior to usage. All other chemicals were purchased from Merck and used as received.

### [Na_2_({SiN^Dipp^}MgOMg{SiN^Dipp^})] (**6**)

In a Young’s tube, [{SiN^Dipp^}MgNa]_2_ (**4**) was dissolved in 0.4 mL of *d*_6_-benzene to make a bright yellow solution. The Young’s tube was kept at ambient temperature over the period of a month, during which time the solution was observed to become pale yellow with the formation of colorless crystals suitable for single-crystal X-ray diffraction analysis at the edge of the solution. X-ray crystallography revealed the identity of the colorless crystals to be **6**.

### [{SiN^Dipp^}Mg(TEMPO)Na] (**7**)

In a Young’s tube, [{SiN^Dipp^}MgNa]_2_ (**4**, 21.6 mg, 0.02 mmol) was dissolved in 0.4 mL of *d*_6_-benzene before the addition of TEMPO (3.1 mg, 0.02 mmol) to the bright yellow solution. The reaction mixture was then kept at room temperature for a period of 3 days, during which time a gradual decolorization and formation of colorless crystals was observed. The colorless crystals were found to be suitable for X-ray diffraction analysis, which established the connectivity of **7**. The colorless crystalline solids were then collected and washed with hexane (0.2 mL × 2) before removal of all volatiles in vacuo, providing **7** as a colorless powder. Yield 16 mg, 65%. No meaningful result was obtained for elemental analysis after multiple attempts. ^1^H NMR (500 MHz, 298 K, chloroform-*d*) δ: 7.06–7.03 (m, 4H, *m*-C_6_*H*_3_), 7.02–6.97 (m, 2H, *p*-C_6_*H*_3_), 3.43–3.30 (m, 4H, C*H*Me_2_), 1.55–1.23 (m, 12H, NC*Me*_2_ of TEMPO), 1.18 (d, *J* = 6.9 Hz, 12H, CH*Me*_2_), 1.15–1.11 (m, 2H, NCMe_2_CH_2_C*H*_2_ of TEMPO), 1.09–1.02 (m, 4H, NCMe_2_C*H*_2_CH_2_ of TEMPO), 0.58 (s, 4H, SiC*H*_2_), 0.08 (s, 12H, SiMe_2_). ^13^C{^1^H} NMR (126 MHz, 298 K, chloroform-*d*) δ: 143.9 (*i*-*C*_6_H_3_), 143.7 (*o*-*C*_6_H_3_), 123.1, 123.0 (*m*-*C*_6_H_3_ and *p*-*C*_6_H_3_), 70.4 (N*C*Me_2_ of TEMPO), 40.0 (NCMe_2_*C*H_2_*C*H_2_ of TEMPO), 31.7 (NCMe_2_CH_2_*C*H_2_ of TEMPO), 28.3 (*C*HMe_2_), 23.7 (CH*Me*_2_), 22.8 (NC*Me*_2_ of TEMPO) 17.4 (NC*Me*_2_ of TEMPO), 9.6 (Si*C*H_2_), −1.6 (Si*Me*_2_).

### [Na_2_({SiN^Dipp^}Mg(C_14_H_10_)Mg{SiN^Dipp^})] (**8**)

In a Young’s tube, [{SiN^Dipp^}MgNa]_2_ (**4**, 21.6 mg, 0.02 mmol) was dissolved in 0.2 mL of *d*_6_-benzene before the addition of anthracene (3.6 mg, 0.02 mmol) to the bright yellow solution. The reaction mixture was then kept at 40 °C for a period of 3 days, exhibiting a gradual decolorization and formation of colorless crystals suitable for X-ray diffraction analysis as **8**. The supernatant was then carefully decanted, and the crystalline solids were collected and washed with hexane (0.1 mL × 2) before removal of all volatiles in vacuo, giving **8** as a colorless powder. Yield 14.5 mg, 58%. All attempts to redissolve **8** in any common solvents (C_6_D_6_, *d*_8_-toluene, *d*_8_-THF, CDCl_3_) induced its degradation and regeneration of free anthracene in the solution. The described process is, however, reproducible with moderate yields of **8** (14.5 mg, 58%; 12.5 mg, 50%; 14 mg, 56%; 15.2 mg, 60%; 14 mg, 56%).

### Reaction of [{SiN^Dipp^}MgNa]_2_ with Benzophenone; Synthesis of [{SiN^Dipp^}Mg(OCPh_2_)_2_] (**9**)

In a Young’s tube, [{SiN^Dipp^}MgNa]_2_ (**4**, 21.6 mg, 0.02 mmol) was dissolved in 0.4 mL of *d*_6_-benzene before the addition of benzophenone (7.2 mg, 0.04 mmol) to the bright yellow solution. The reaction mixture instantaneously turned into a purple solution. The reaction mixture was then left standing at room temperature overnight, whereupon the formation of orange precipitates was observed. The purple supernatant was then carefully decanted and examined by EPR spectroscopy. The orange solid was collected and redissolved in toluene, and bright yellow crystals suitable for single-crystal X-ray diffraction were obtained by storage of the orange solution at −30 °C. This reaction was then repeated with a modified stoichiometry of substrates: [{SiN^Dipp^}MgNa]_2_ (**4**, 21.6 mg, 0.02 mmol) was dissolved in 0.4 mL of *d*_6_-benzene before the addition of benzophenone (21.6 mg, 0.12 mmol) to the bright yellow solution. This entry also provided a purple solution with orange solids when the reaction mixture was kept at ambient temperature overnight. The purple supernatant provided a virtually identical EPR spectrum, and the recrystallization of the orange solids provided compound **9** (confirmed by unit-cell screening). Yield 6.9 mg, 19%. Compound **9** can also be prepared by treatment of 2 equiv of benzophenone (7.2 mg, 0.04 mmol) with [{SiN^Dipp^}Mg] (10.4 mg, 0.02 mmol).^33a^ The reaction was conducted with 0.4 mL of C_6_D_6_ inside a J-Young’s tube, providing quantitative conversion into **9** (verified by NMR spectroscopy), and slow evaporation of the benzene solution provided **9** as bright yellow crystals. Yield 15.2 mg, 86%. No meaningful result was obtained for elemental analysis after multiple attempts. ^1^H NMR (500 MHz, 298 K, benzene-*d*_6_) δ: 7.19–7.16* (m, 8H, Ar*H*,*overlapping with C_6_D_6_), 7.14–7.00 (m, 11H, Ar*H*), 6.95–6.92 (m, 7H, Ar*H*), 4.31 (sept, *J* = 6.9 Hz, 4H, CH*Me*_2_), 1.44 (s, 4H, SiC*H*_2_), 1.41 (d, *J* = 6.9 Hz, 12H, CH*Me*_2_), 0.89 (d, *J* = 6.9 Hz, 12H, CH*Me*_2_), 0.38 (s, 12H, Si*Me*_2_). ^13^C NMR (126 MHz, 298 K, Benzene-*d*_6_) δ: 151.6 (*i*-*C*_6_H_3_), 151.3 (*i*-*C*_6_H_5_ on OCPh_2_), 145.6 (*o*-*C*_6_H_3_), 133.5 (*o*-*C*_6_H_5_ on OCPh_2_), 131.3 (*p*-*C*_6_H_5_ on OCPh_2_), 128.6 (*m*-*C*_6_H_5_ on OCPh_2_), 123.5 (*m*-*C*_6_H_3_), 120.3 (*p*-*C*_6_H_3_), 114.1 (O*C*Ph_2_), 27.5 (*C*HMe_2_), 25.3 (CH*Me*_2_), 24.3 (CH*Me*_2_), 13.4 (Si*C*H_2_), 1.4 (Si*Me*_2_).

### [Na_2_({SiN^Dipp^}Mg(PhCCPh)Mg{SiN^Dipp^})] (**10**)

In a Young’s tube, [{SiN^Dipp^}MgNa]_2_ (**4**, 21.6 mg, 0.02 mmol) was dissolved in 0.4 mL of *d*_8_-toluene before the addition of diphenylacetylene (3.6 mg, 0.02 mmol) to the bright yellow solution. The reaction mixture was then kept at 40 °C for a period of 3 days, whereupon the ^1^H NMR spectrum indicated the formation of a new predominant species and the consumption of all starting materials. The tube was then taken into the glovebox, and the now yellowish-orange solution was decanted into a vial. Slow evaporation of the solution provided **10** as pale orange crystals suitable for X-ray diffraction analysis. Yield 17 mg, 67%. No meaningful result was obtained for elemental analysis after multiple attempts. ^1^H NMR (500 MHz, 298 K, toluene-*d*_8_) δ: 7.36–7.26 (m, 2H, C_6_*H*_3_ on SiN^Dipp^), 7.26–7.18 (m, 5H, C_6_*H*_5_ on PhCCPh), 6.87–6.49 (m, 10H, C_6_*H*_3_ on SiN^Dipp^), 6.60–6.50 (m, 5H, C_6_*H*_5_ on PhCCPh), 4.15–3.65 (m, 4H, C*H*Me_2_) 3.65–3.02 (m, 4H, C*H*Me_2_), 1.38–1.05 (m, 30H, CH*Me*_2_), 1.05–0.65 (m, 18H, CH*Me*_2_), 0.65–0.44 (m, 8H, Si*C*H_2_), 0.35 – −0.05 (m, 24H, Si*Me*_2_). ^13^C{^1^H} NMR (101 MHz, 298 K, toluene-*d*_8_) δ: 156.9 (4° *C* of C_6_H_3_ on SiN^Dipp^), 153.6 (4° *C* of C_6_H_3_ on SiN^Dipp^), 144.3 (Ar*C* of PhCCPh), 139.8 (A r*C* of PhCCPh), 132.1 (4° *C* of PhCCPh), 131.9 (Ar *C* of C_6_H_3_ on SiN^Dipp^), 126.9 (4° *C* of PhCCPh), 123.3 (Ar *C* of C_6_H_3_ on SiN^Dipp^), 119.4 (Ar *C* of PhCCPh), 28.5 (CH*Me*_2_), 27.3 (*C*HMe_2_), 23.6 (CH*Me*_2_), 9.8 (Si*C*H_2_), −1.6 (Si*Me*_2_).

### EPR Spectroscopy

Samples for EPR measurements were loaded into Young’s EPR tubes under an N_2_ atmosphere in a glovebox. The X-band CW EPR measurements (298 K) were performed on a Bruker EMX spectrometer utilizing an ER 072 magnet/ER 081 power supply combination (maximum field 0.6 T), an ER4119HS resonator, operating at 100 kHz modulation frequency and 0.5 G modulation depth (no improvement in resolution of hyperfine features was observed by implementing 10 kHz modulation frequency with 0.1 G modulation depth) and 10 mW microwave power. Simulations of all EPR spectra were performed using the garlic function within the EasySpin toolbox for Matlab.^37^

## Results and Discussion

### *O*-Centered Oxidants: Dioxygen and TEMPO

**Scheme 1:**
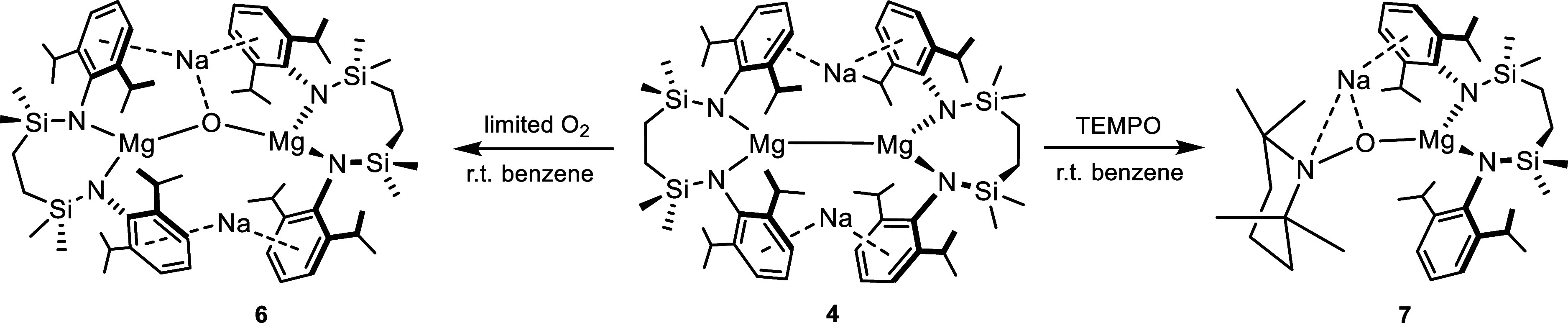
Oxidation of **4** to Provide Compounds **6** and **7**

**Figure 2:**
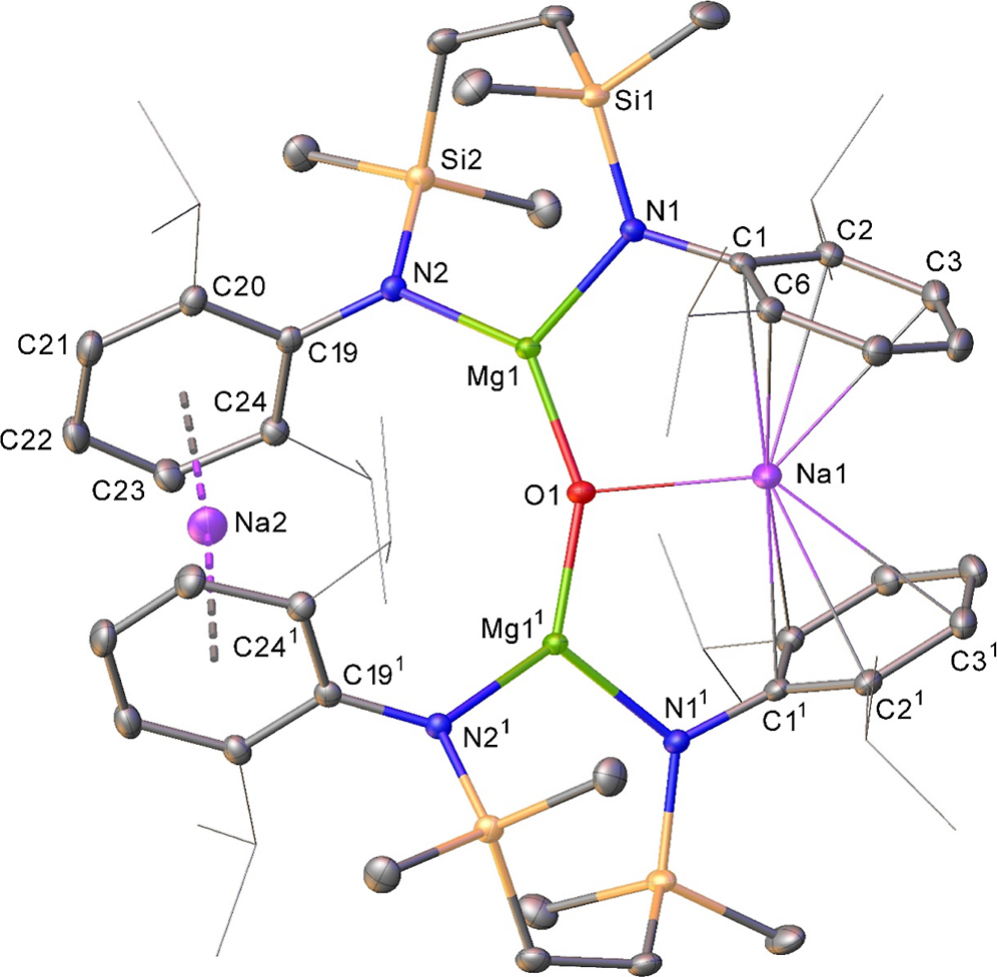
Displacement ellipsoid (30% probability) plot of compound **6**. Hydrogen atoms and solvents have been removed for the sake of clarity. Additionally, *iso*-propyl groups are shown as wireframes, also for visual ease. Selected bond lengths (Å) and angles (deg): Mg1–O1 1.8610(4), Mg1–N1 2.0366(9), Mg1–N2 2.0537(9), Na1–O1 2.2481(12), Na1–C1 2.7477(9), Na1–C2 2.7800(10), Na2–C19 2.8470(10), Na2–C21 2.7542(12), Na2–C22 2.6999(13), Na2–C23 2.6575(12), O1–Mg1–N1 114.62(4), O1–Mg1–N2 129.48(4), N1–Mg1–N2 114.89(4). Symmetry operations generated equivalent atoms ^1^ 1/2 – *x*, *y*, 1 – *z*.

Initial serendipitous insight into the oxidation of compound **4** was provided by storage of a benzene solution in a vessel fitted with a Young’s valve. A small batch of colorless crystals, which were deposited at room temperature over the course of several weeks, were shown by X-ray diffraction analysis to be [Na_2_({SiN^Dipp^}MgOMg{SiN^Dipp^})] (**6**, Scheme 1), apparently due to ingress of adventitious dioxygen and where a now doubly reduced oxygen atom bridges between two magnesium centers (Figure 2). The N–Mg distances in **6** (avg. 2.027 Å) are shorter than those in **4** (avg. 2.083 Å), supporting the higher 2+ oxidation state assigned to magnesium. These bond lengths are, however, significantly elongated in comparison to the N–Mg bonds found in the Mg(II) precursor to compound **4**, [{SiN^Dipp^}Mg(benzene)] (avg. 1.9454 Å),^33a^ presumably a consequence of the stronger *O*-donation provided by the two benzophenone ligands to the magnesium centers. The Mg–O bond [1.8610(4) Å] in compound **6** is slightly longer than those in the dimagnesium-oxo-complex obtained from the nitrous oxide oxidation of [{^Dipp^BDI}Mg]_2_ (**2**) (Mg–O, 1.8080(5); Mg–N, avg. 2.104 Å), despite the isolation of the previously reported molecule as a THF adduct.^38^

Subsequent attempts to achieve a more rational and higher-yielding synthesis of heterobimetallic oxide **6** with limited amounts of either molecular oxygen or N_2_O were unsuccessful, presumably due to the indiscriminate nature of these elemental and molecular oxidants. Treatment of **4** with a stoichiometric equivalent of the *O*-centered oxidant (2,2,6,6-tetramethylpiperidin-1-yl)oxyl (TEMPO), however, provided smooth access to a single new heterobimetallic compound (**7**, Scheme 1). A gradual decolorization was observed upon the addition of the TEMPO radical to the bright yellow *d*_6_-benzene solution of **4**, and, on standing overnight at a slightly elevated temperature (40 °C), the reaction mixture deposited colorless single crystals. Although the limited solubility of the isolated crystals hampered further characterization by NMR spectroscopy in C_6_D_6_, X-ray diffraction analysis revealed the outcome of the reaction to be [({SiN^Dipp^}Mg)(TEMPO)Na] (**7**), the one-to-one product of TEMPO single electron reduction by each dimer half of the [{SiN^Dipp^}MgNa]_2_ molecule. Although the solid-state data for compound **7** were of insufficient quality (*R*_1_ = 0.0792, *wR*_2_ = 0.2153) to encourage more precise commentary, they unambiguously verified the facility of each [{SiN^Dipp^}MgNa] dimer half of **4** to effect single electron reduction (Figure S1). Increased solubility in the more polar solvent, CDCl_3_, did allow NMR spectroscopic characterization of compound **7**. A symmetrical disposition across the {SiN^Dipp^}-backbone in the molecule could be inferred from its ^1^H NMR spectrum, in which the diagnostic *iso*-propyl methine (δ 3.43–3.30 ppm, 4H) and SiMe_2_ (δ 0.08 ppm 12H) signals were each observed to resonate as single environments. This observation is consistent with a time-averaged *C*_2_-symmetric conformation across the molecule originating from the loss of the persistent Mg–Mg interactions between the sodium-bridged [{SiN^Dipp^}Mg] moieties of compound **4**.^33a^

### Anthracene and Benzophenone

**Scheme 2:**
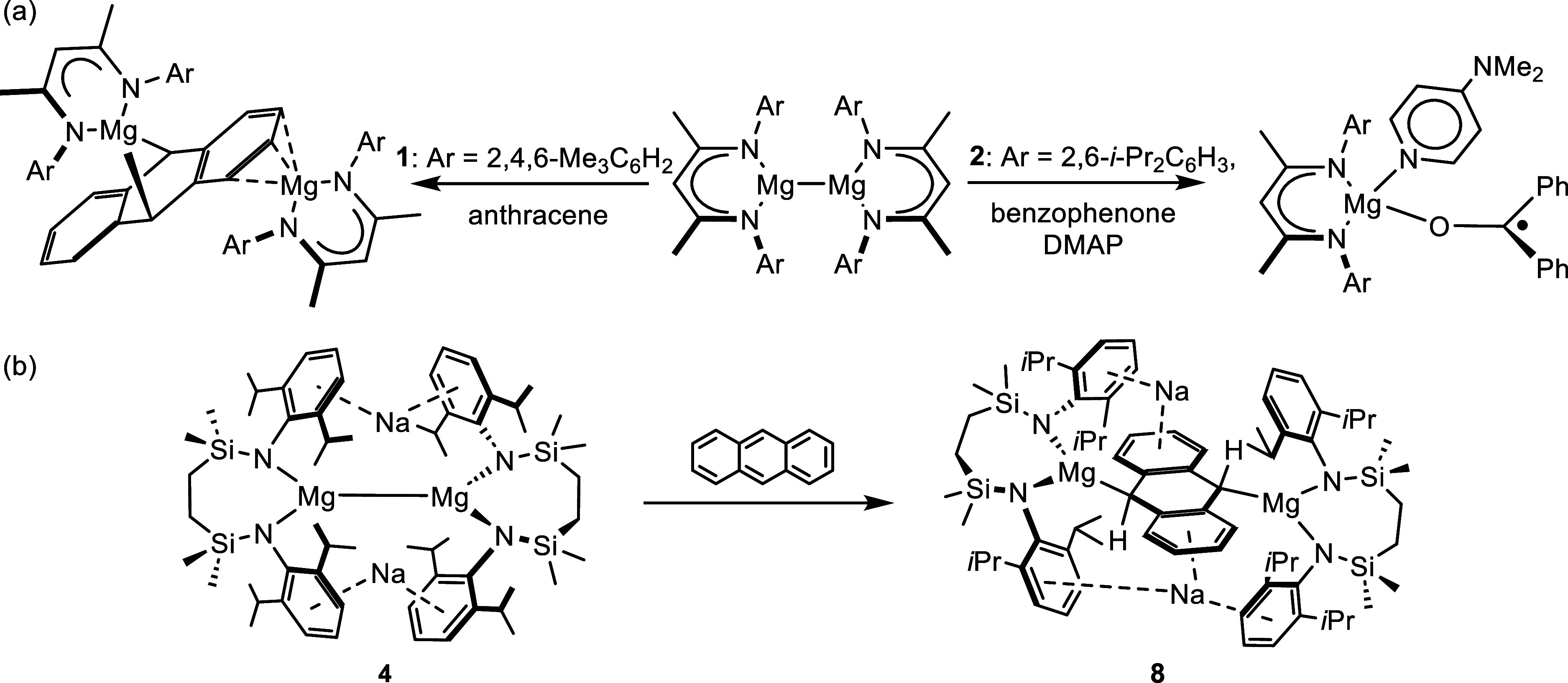
(a) Previously Reported Reactivity of Compounds **1** and **2** toward Anthracene and Benzophenone;^29^ (b) Synthesis of Compound **8**

**Figure 3:**
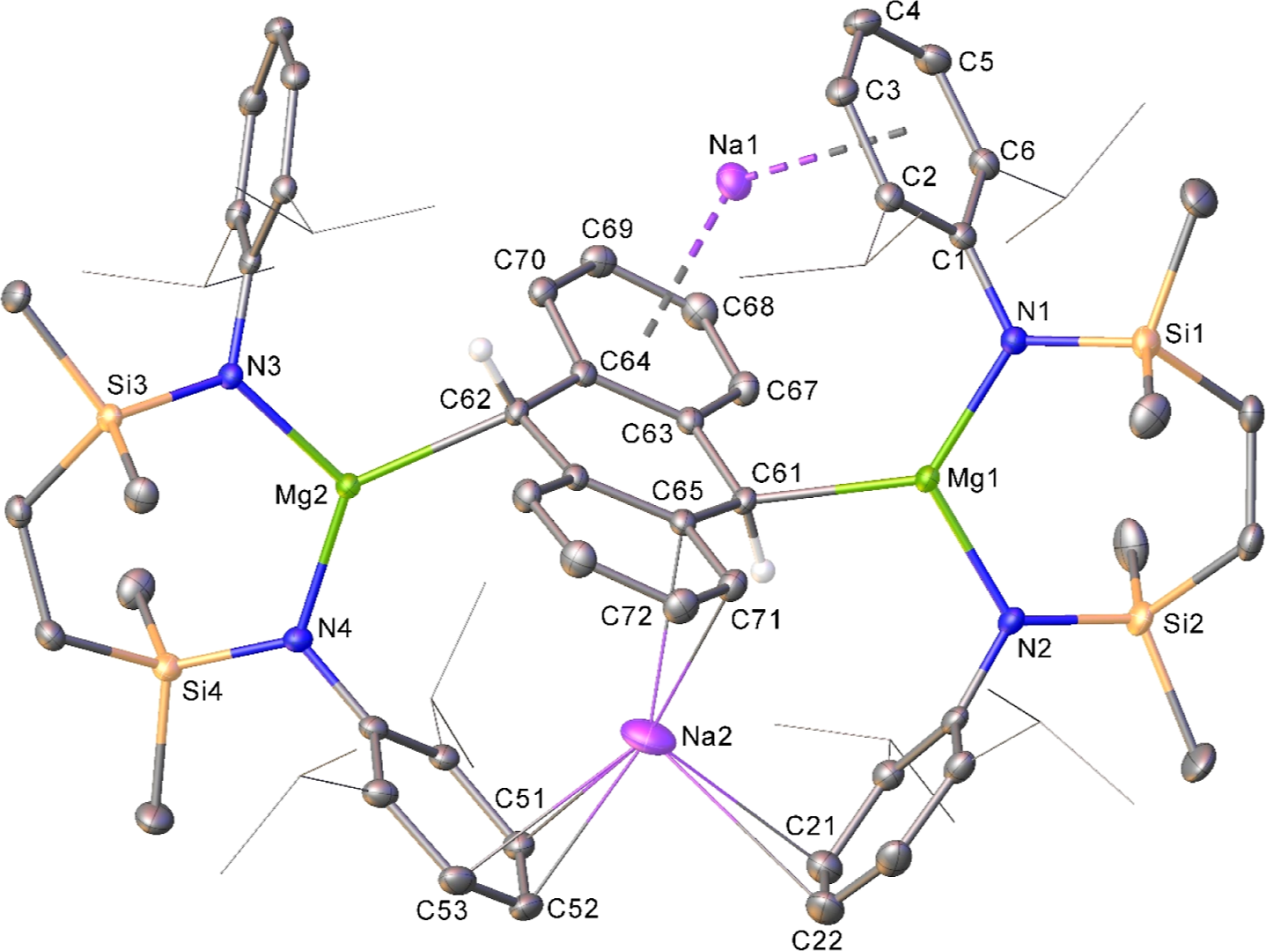
Displacement ellipsoid (30% probability) plot of compound **8**. Hydrogen atoms (C61 and C62 excepted) have been removed, and *iso*-propyl groups are shown as wireframes, for clarity. Selected bond lengths (Å) and angles (deg): Mg1–N1 1.9990(12), Mg1–N2 2.0098(12), Mg1–C61 2.2342(13), Mg2–N3 2.0043(11), Mg2–N4 2.0099(12), Mg2–C62 2.2408(13), Na1–C2 2.9186(15), Na1–C3 2.7217(16), Na1–C4 2.6730(18), Na1–C67 2.8703(16), Na1–C68 2.8171(17), Na1–C69 2.7530(16), Na1–C70 2.7824(15), N1–Mg1–N2 117.98(5), N3–Mg2–N4 117.26(5).

An early demonstration of the reducing nature of the β-diketiminato compounds **1** and **2** was provided by Jones and co-workers’ study of their behavior toward anthracene and benzophenone, which provided the respective deep red bimetallic and purple monometallic and radical products of reduction (Scheme 2a).^29,39^ Compound **4** was, thus, reacted with anthracene (*E*^0^ = −1.98 V vs SCE)^40^ in C_6_D_6_ at 40 °C for a period of 3 days. This procedure resulted in the gradual decolorization of the solution and the formation of colorless single crystals of compound **8**. Although all subsequent attempts to redissolve **8** in any common solvents (C_6_D_6_, *d*_8_-toluene, *d*_8_-THF, CDCl_3_) resulted in its decomposition and the regeneration of free anthracene as the only identifiable species in solution, X-ray diffraction analysis afforded its solid-state identification (Figure 3). Compound **8** is a heterotetrametallic species in which, although noncentrosymmetric, two [{SiN^Dipp^}Mg] units interact via similar κ^1^-engagement with the central C_6_-carbocycle, albeit across opposing faces of a doubly reduced anthracenyl dianion. Consistent with a formal Mg(II) oxidation state, the Mg–N separations (average 2.006 Å) are notably shorter than those in compound **4** (average 2.083 Å). Consistent with these assignments, the C61–C63–C64–C62–C66–C65 carbocycle is best denoted as a 1,4-cyclohexadienyl structure with alternating long and short C–C bond distances [C61–C63 1.4895(19) Å, C63–C64 1.4277(18) Å, C64–C62 1.4751(18) Å, C62–C66 1.4795(18) Å, C66–C65 1.4259(18) Å, C65–C61 1.4958(18) Å], wherein the only significant contacts to magnesium are provided by the now pseudotetrahedral C61 and C62 methine carbon atoms [Mg1–C61 2.2342(13); Mg2–C62, 2.2408(13) Å]. The structure is completed by polyhapto encapsulation of Na1 and Na2 on opposing faces of the anthracenyl dianion, in each case through a peripheral C_6_ carbocycle and a single Dipp substituent of each [{SiN^Dipp^}Mg] moiety. This influential structural feature ensures that the disodium anthracenyl bis-magnesiate structure of **8** contrasts significantly with Jones’ derivative in which the cationic β-diketiminato magnesium units displayed contrasting modes of engagement with the polycyclic arene dianion (Scheme 2a).

**Figure 4:**
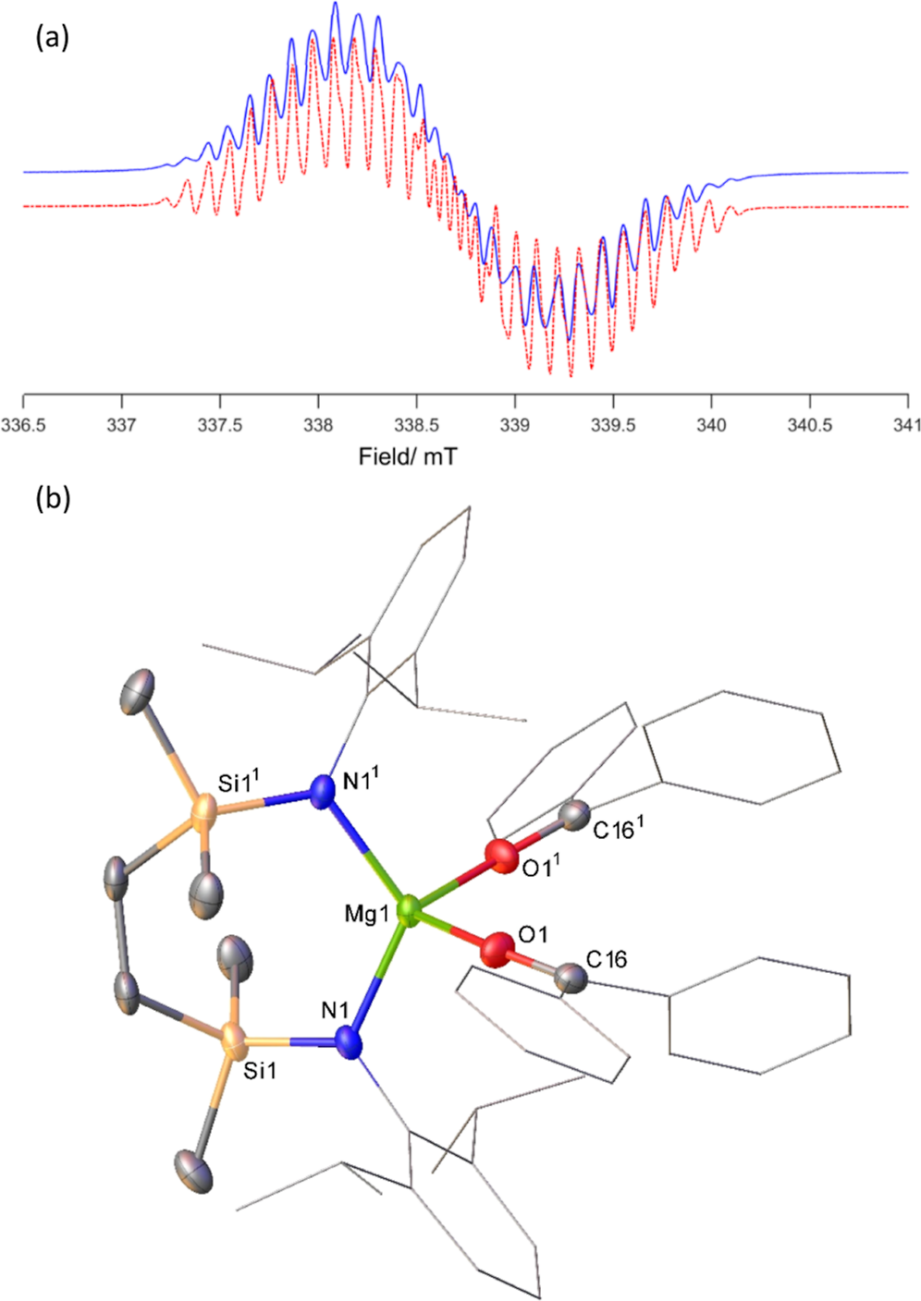
(a) EPR signal (blue solid) and simulation (red dashed) of the purple supernatant solution provided by the reaction of compound **4** and benzophenone (see ESI for details); (b) displacement ellipsoid (30% probability) plot of compound **9**. Hydrogen atoms have been removed, while Dipp substituents and phenyl groups are shown as wireframes for clarity. Selected bond lengths (Å) and angles (deg): Mg1–O1 2.0138(16), Mg1–O1^1^ 2.0137(16), Mg1–N1 2.0135(18), Mg1–N1^1^ 2.0135(18), N1–Mg1–O1 103.97(7), N1–Mg1–O1^1^ 109.98(7), N1^1–^Mg1–O1^1^ 103.97(7), N1–Mg1–N1^1^ 121.70(11). Symmetry operations generated equivalent atoms ^1^1 – *x*, *y*, 1/2 – *z*.

Treatment of a benzene solution of compound **4** with benzophenone resulted in the immediate generation of a purple solution and the precipitation of an orange crystalline material. A similar result was obtained irrespective of the reaction stoichiometry, while filtration and analysis of the solutions by NMR spectroscopy provided spectra that were broad and unassignable. Cognizant of likely ketyl radical formation, the purple supernatant was analyzed by EPR spectroscopy. While it is plausible that a component of this solution comprised bimetallic aggregates that include both magnesium and sodium, the resultant spectrum and simulation provided parameters that were redolent of a sodium ketyl with little, if any, perturbation to the ketyl SOMO arising from the presence of any [{SiN^Dipp^}Mg] component in solution (Figure 4a).^41^ This inference that the dissimilar alkaline metals behave with mutual independence subsequent to ketone addition was further supported by recrystallization of the orange precipitate, which allowed the identification of the resultant bright yellow crystals of compound **9** by X-ray diffraction analysis as a bis-ligated benzophenone adduct of [{SiN^Dipp^}Mg] (Figure 4b). While the rational synthesis (86%) and complete characterization of compound **9** were readily achieved by treatment of [{SiN^Dipp^}Mg] with 2 equiv of benzophenone, the structure is unremarkable. Its identification, however, demonstrates both the facility for the oxidation of the Mg(I) centers of compound **4** by benzophenone and the solution lability of any consequent Na–O- and Mg–O-bound species, irrespective of the oxidation level of the ketone reagent.

### Alkenes and Alkynes

**Figure 5:**
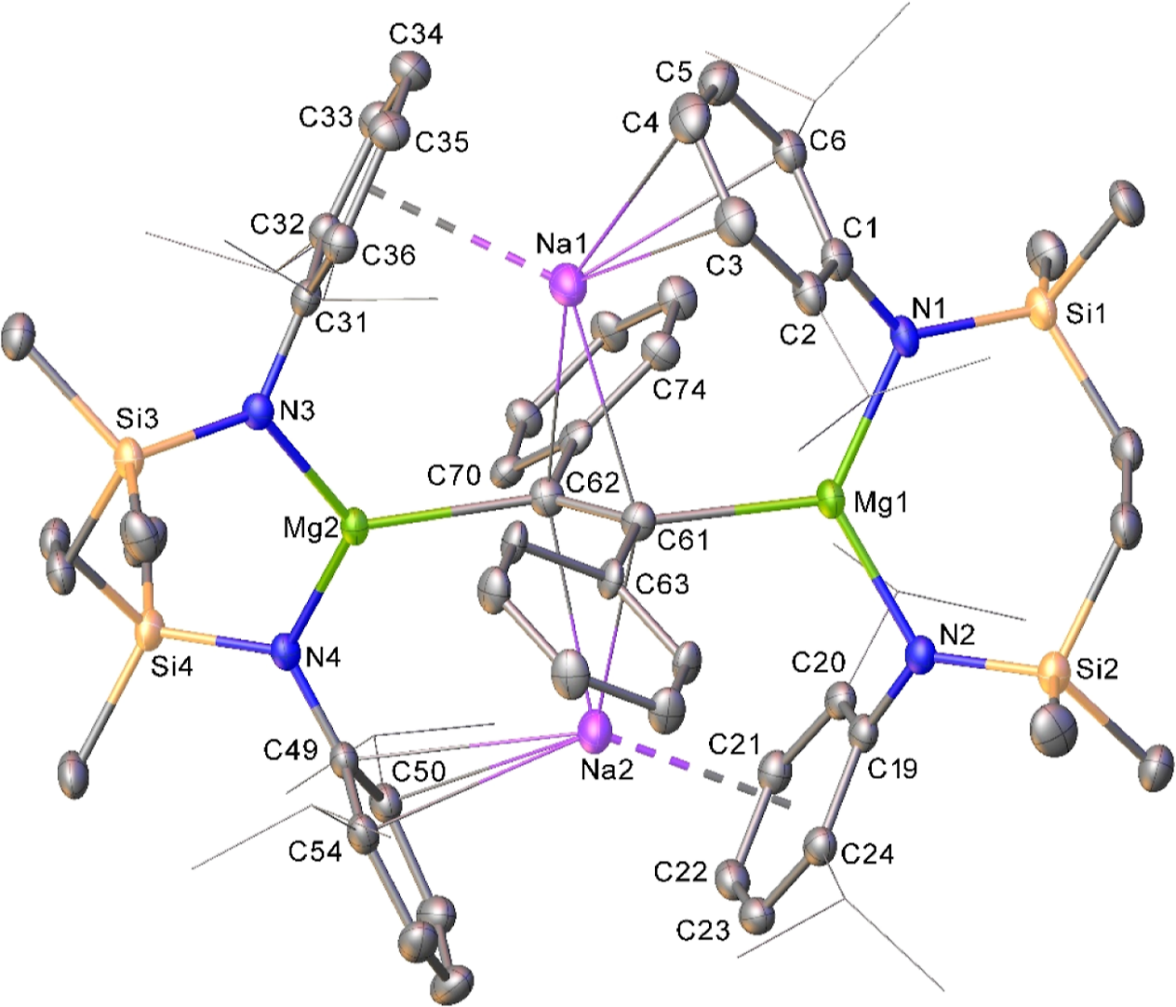
Displacement ellipsoid (30% probability) plot of compound **10**. Hydrogen atoms, the minor disordered component atoms, and the solvent have been removed for clarity. In addition, *iso*-propyl groups are shown as wireframes for visual ease. Selected bond lengths (Å) and angles (deg): Mg1–N1 2.049(3), Mg1–N2 2.057(3), Mg1–C61 2.221(4), Mg2–N3 2.043(3), Mg2–N4 2.038(3), Mg2–C62 2.228(4), Na1–C61 2.872(6), Na1–C62 2.819(6), Na2–C61 2.867(6), Na2–C62 2.803(7), and C61–C62 1.370(7).

Further guided by Jones and co-workers’ wide-ranging studies of **1** and **2**,^42,43^ solutions of compound **4** were treated with a selection of alkenes and alkynes. While no evidence of reaction was observed with either terminal or internal alkenes such as styrene, 1,1-diphenylethylene, or *trans*-stilbene, even when heated to the decomposition point of the bimetallic compound, treatment with diphenylacetylene at 40 °C for 3 days resulted in consumption of the starting materials and the formation of a single predominant product, compound **10**. Consistent with significant solution lability, the ^1^H NMR spectrum of compound **10** in *d*_8_-toluene exhibited broad resonances, which, although assignable, provided only limited structural insight. While all attempts to change the solvent resulted in the decomposition of the molecule with the formation of protonated [{SiN^Dipp^}H_2_] as the only assignable species, single crystals of **10** were obtained by slow evaporation of a toluene solution at low temperature. The resultant X-ray diffraction analysis identified compound **10** as a 1,2-dimagnesioethene species (Figure 5), in which a {PhC=CPh}^2–^ dianion [C61–C62 1.370(7) Å] bridges two symmetrically disposed [{SiN^Dipp^}Mg] moieties. The structure is completed by two arene-encapsulated sodium cations, which balance the overall charge of the molecule and reside with a symmetrical disposition on opposing sides of the ethene diide moiety [Na1–C61 2.872(6) Å; Na1–C62 2.819(6) Å; Na2–C61 2.867(6) Å; Na2–C62 2.803(7) Å].

Although the most relevant precedent for the synthesis of compound **10** is provided by Jones and co-workers’ report of the reactivity of compound **1** with diphenylacetylene,^43^ alkyne reduction with magnesium vapor has also been reported to provide the tetrameric aggregate, [{Mg(THF)C(Ph)C(Ph)}_4_], in which the {PhC=CPh}^2–^ units were formed as a mixture of *E*- and *Z*-isomers.^44,45^ In contrast, and in a manner similar to Jones’ β-diketiminate-supported precedent, the {PhC=CPh}^2–^ dianion of **10** displays an exclusive *trans*-disposition. While Jones and co-workers ascribed this notable regioselectivity as a presumed consequence of steric/kinetic considerations, the more constrained constitution of compound **4** raises pertinent mechanistic questions with regard to the mode of delivery and reduction of the organic substrate during its transformation to compound **10**.

Intrigued by the stereospecific outcome of diphenylacetylene reduction with **4**, density functional theory (DFT) calculations were performed at the BP86-D3BJ(PCM = Benzene)/BS2//BP86/BS1 level of theory. Initial assessment of the two possible geometric isomers revealed that the formation of the *E*-{PhC=CPh}^2–^ product, **P**_**E**_ (−57.4 kcal mol^–1^), is more thermodynamically favored than the *Z*-{PhC=CPh}^2–^ product, **P**_**Z**_ (−27.9 kcal mol^–1^). Mechanisms invoking either direct *anti*- (necessary for the direct formation of **P_**Z**_**) or *syn*-addition (i.e., prior to *Z* → *E* isomerization) of PhC≡CPh were, thus, considered. In neither case could, however, any viable direct interaction of diphenylacetylene with the sterically encumbered {Mg_2_Na_2_} core be identified. Although this implies that the SiN^Dipp^ ligands afford too limited access for the direct addition of more bulky unsaturated species, prior studies of **4** have revealed the {SiN^Dipp^Mg}_2_ chelate to be somewhat labile and amenable to macrocyclization.^35^

**Figure 6:**
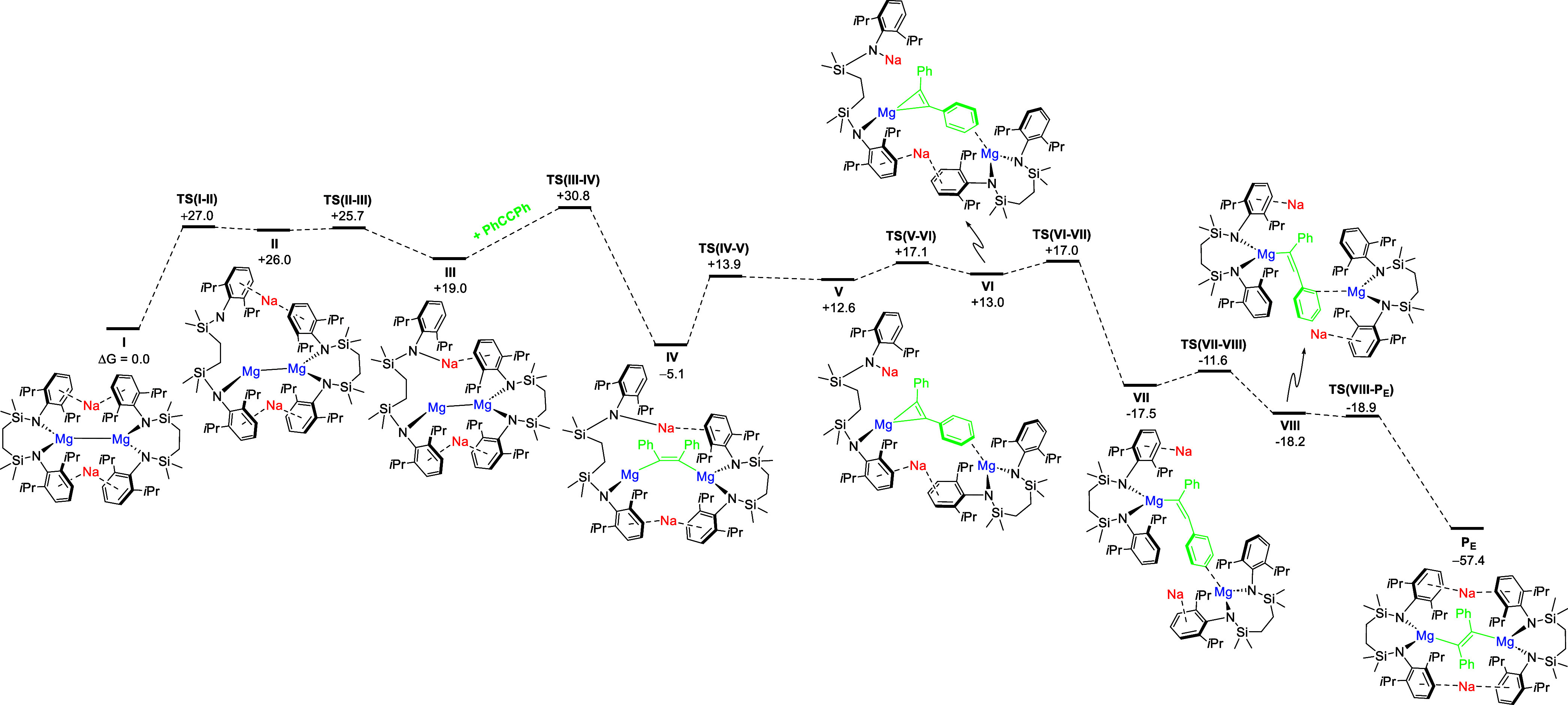
Free energy profile (calculated with DFT at the BP86-D3BJ(PCM = Benzene)/BS2//BP86/BS1 level of theory, energies in kcal mol^–1^) of addition and reduction of diphenylacetylene at **I** to form **P**_**E**_.

**Figure 7:**
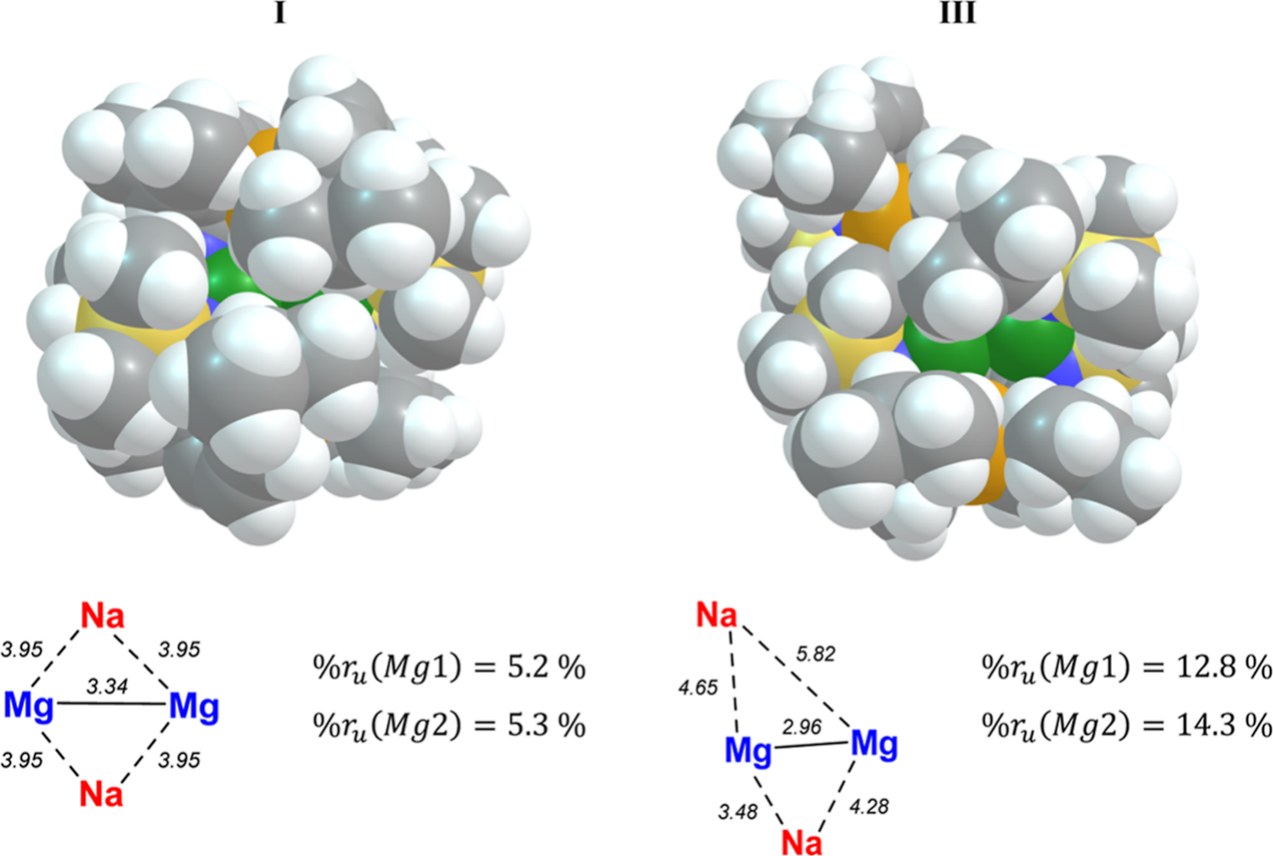
Structure with vdW spheres, intermetallic distances (Å) within the {Mg_2_Na_2_} tetrad, and % unblocked rays (%*r*_u_) of Mg centers in **I** (left) and **III** (right).

A mechanism involving amide ligand cleavage at either or both magnesium centers was thus considered as a means to alleviate the steric constraints about the {Mg_2_Na_2_} core. Accordingly, Mg–N cleavage was successfully characterized through a two-step Mg^+^ → Na^+^ amido group migration to form **III** (+19.0 kcal mol^–1^, Figure 6). The thermodynamic feasibility of such Mg^+^ → Na^+^ migration appears consistent with available bond dissociation energies of Mg^+^ or Na^+^ with ammonia, which indicate comparable H_3_N → M^+^ interaction strengths between the two *s*-block cations.^46,47^ Moreover, this process both desymmetrizes the {Mg_2_Na_2_} tetrad and results in a lower shielding of the Mg(I) centers by the encapsulating Dipp groups, as indicated by calculation of the percentage of “unblocked” rays (%*r*_u_) from each Mg center via a raytracing algorithm designed to quantify and predict the steric accessibility of a specified atomic center in a given molecular system (Figure 7).^48^ With this metric, a step-change in the “accessibility” of each Mg(I) center is quantified between **I** [%*r*_u_ (Mg1,Mg2) = 5.2%] and **III** [%*r*_u_ (Mg1,Mg2) = 12.8%, 14.3%].

From **III**, *syn*-addition of diphenylacetylene could now be characterized via an accessible saddle point, **TS(III–IV)** (+30.8 kcal mol^–1^), forming *Z*-{PhC = CPh}^2–^ adduct **IV** (−5.1 kcal mol^–1^). Subsequent *Z* → *E* isomerization was then postulated to take place prior to ring-closure and ultimate formation of **10**/**P**_**E**_. This is initiated by Mg–C cleavage (via **TS(IV–V)**, +13.9 kcal mol^–1^, ΔΔ*G*^⧧^ = +19.0 kcal mol^–1^ relative to **IV**), forming magnesacyclopropenyl{MgC_2_Ph_2_}^2–^ adduct **V**, (+12.6 kcal mol^–1^, *r*_Mg–C1_ = 2.09 Å, *r*_Mg–C1_ = 2.18 Å, *r*_C1=C2_ = 1.39 Å), whereby the flanking Mg^II^ center interacts with a sp^2^-C atom at the Ph group. Subsequent migration of this flanking Mg^II^ center to the opposite aryl face affords **VI** (+13.0 kcal mol^–1^), prior to ring-closing amido group migration from Na^+^ → Mg^+^ via **TS(VI–VII)**, +17.6 kcal mol^–1^, which results in a concerted opening of the strained magnesacyclopropenyl ring to form linear vinylmagnesium adduct **VII** (−17.5 kcal mol^–1^). This is followed by migration of the other SiN^Dipp^Mg^II^ group via **TS(VII–VIII)** (−18.9 kcal mol^–1^), where Mg–C formation through **TS(VIII–P**_**E**_**)**, −18.9 kcal mol^–1^, yields the ultimate *E*-{PhC=CPh}^2–^ product **P**_**E**_ (−57.4 kcal mol^–1^). Consistent with the slow process that is experimentally observed even at elevated temperatures (3 days, 40 °C), the largest energetic span overcome during the transformation is, thus, +30.8 kcal mol^–1^. Finally, Atoms in Molecules analysis of the resulting product **P**_**E**_ (**10**) supports the formal assignment of an *E*-{PhC=CPh}^2–^ dianion, with significant charge localization at the alkenyl carbon atoms (*r*_C1=C2_ = 1.39 Å, ε_C1=C2_ = +0.152, q_C1,C2_ = −0.62), encapsulated within a tetrad of Mg^2+^ and Na^+^ cations (q_Mg1,Mg2_ = +1.63, q_Na1,Na2_ = +0.89).

## Conclusions

Although our observations of its reactivity with benzophenone imply that the group 1 and group 2 centers within compound **4** do hold the potential to behave independently, the majority of redox processes observed thus far have resulted in bimetallic products. While such empirical studies can provide only circumstantial evidence of bimetallic cooperativity, the computational investigation of PhC≡CPh addition has revealed a previously uncharacterized and rather unusual mode of reactivity. While also providing insight into the otherwise unaccounted for amenability toward macrocyclization of the bidentate SiN^Dipp^ ligand, this analysis invokes a series of elementary steps which necessitate (a) ring-opening via Mg^+^ → Na^+^ amido group migration of the SiN^Dipp^ ligand, (b) *syn*-addition of diphenylacetylene to form a *Z*-{PhC=CPh}^2–^ dianion, (c) *Z* → *E* isomerization of the {PhC=CPh}^2–^ group, and (d) ultimate ring-closure via Na^+^ → Mg^+^ amido group migration. The pairing of the {Mg_2_Na_2_} core with the labile SiN^Dipp^ ligands may, therefore, enable direct reductions of unsaturated substrates that are too cumbersome for other conventional *s*-block reductants through this unusual ring opening process. We continue to explore this hypothesis with **4** and related compounds.

## Supporting Information Available

General procedures, NMR spectra, crystallographic data, and details of the computational analysis (PDF)
Cartesian coordinates of all computed structures available in the xyz format (XYZ)

## Supplementary Material

om4c00076_si_001.pdf

om4c00076_si_002.xyz
