# Supplementary material for: [{SiNDipp}MgNa]2: A Potent Molecular Reducing Agent
Source: Organometallics. 2024 Apr 9;43(8):879–88. doi: 10.1021/acs.organomet.4c00076 (PMC11041119; doi:10.1021/acs.organomet.4c00076)
Supplement: Supplementary file 1 — om4c00076_si_001.pdf [file om4c00076_si_001.pdf]

Supporting Information for

$[(SiN^{Dipp})MgNa]_2$: a Potent Molecular Reducing Agent

Han-Ying Liu,[a] Samuel E. Neale,[a] Michael S. Hill,[a]* Mary F. Mahon,[a] Claire L. McMullin[a]* and Emma Richards[b]

[a] *Department of Chemistry, University of Bath, Claverton Down, Bath, BA2 7AY*

[b]*School of Chemistry, Cardiff University, Main Building, Park Place, Cardiff, CF10 3AT.*

1 Experimental and Supplementary Information

1.1. General information

Unless stated otherwise, all the experiments were conducted using standard Schlenk line and/or glovebox techniques under an inert atmosphere of argon. NMR spectra were recorded with an Agilent ProPulse spectrometer (^{1}H at 500 MHz, ^{13}C at 126 MHz). The spectra are referenced relative to residual protio solvent resonances. Elemental analyses were performed at Elemental Microanalysis Ltd., Okehampton, Devon, UK. Solvents were dried by passage through a commercially available solvent purification system and stored under argon in ampoules over 4 Å molecular sieves. C_6D_6 was purchased from Sigma-Aldrich, dried over a potassium mirror before distilling and storage over molecular sieves. $[\{SiN^{Dipp}\}MgNa]_2$ (**4**),[1] was prepared according to reported procedures. All other chemicals were purchased from Sigma-Aldrich Merck and used without further purification.

Figure S1: Connectivity of compound **7** established by a single crystal X-ray diffraction study.

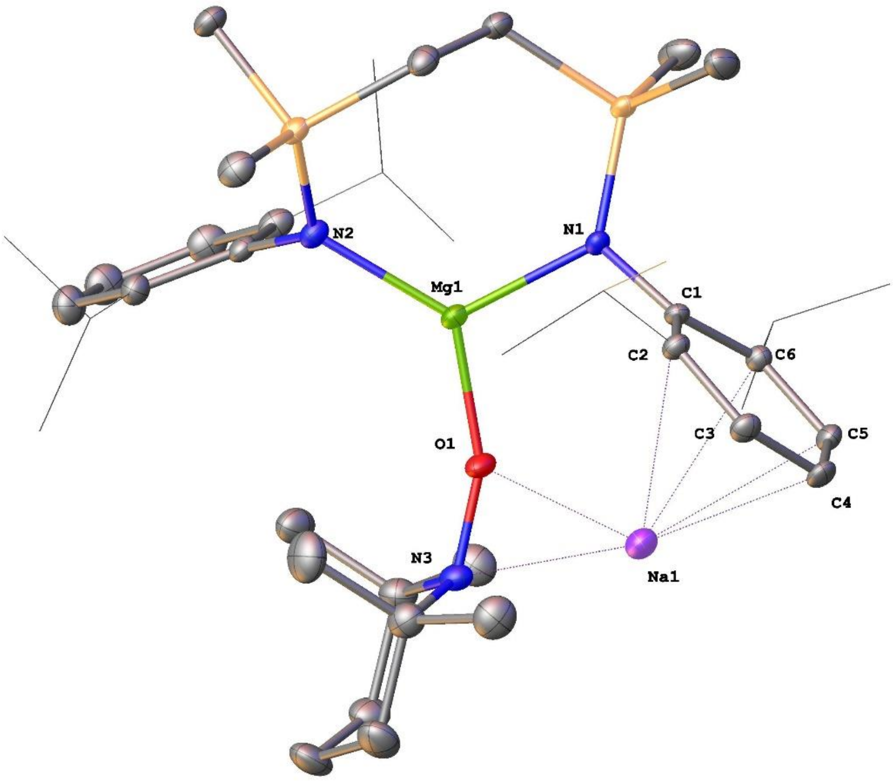

Figure S2: ^{1}H NMR spectrum (500 MHz, 298 K, $CDCl_3$) of compound **7**.

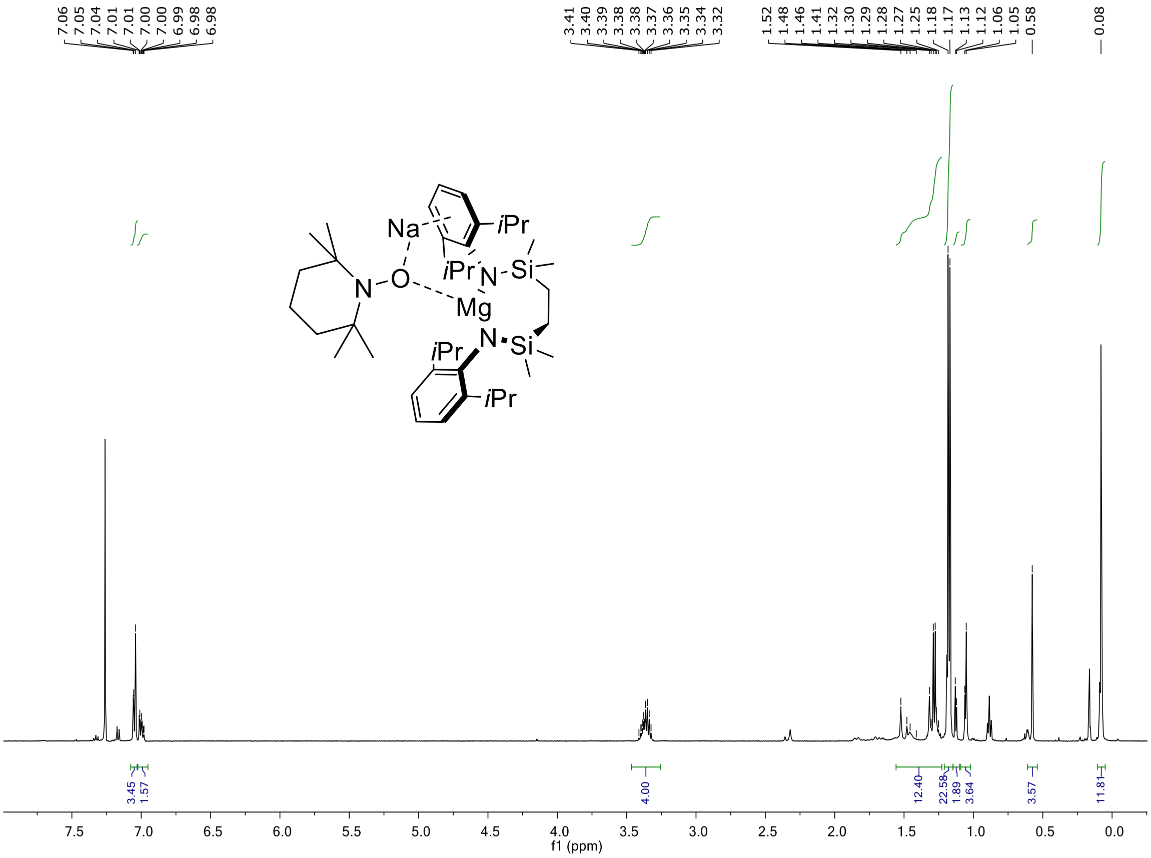

Figure S3: $^{13}C\{^{1}H\}$ NMR spectrum (126 MHz, 298 K, $CDCl_3$) of compound **7**.

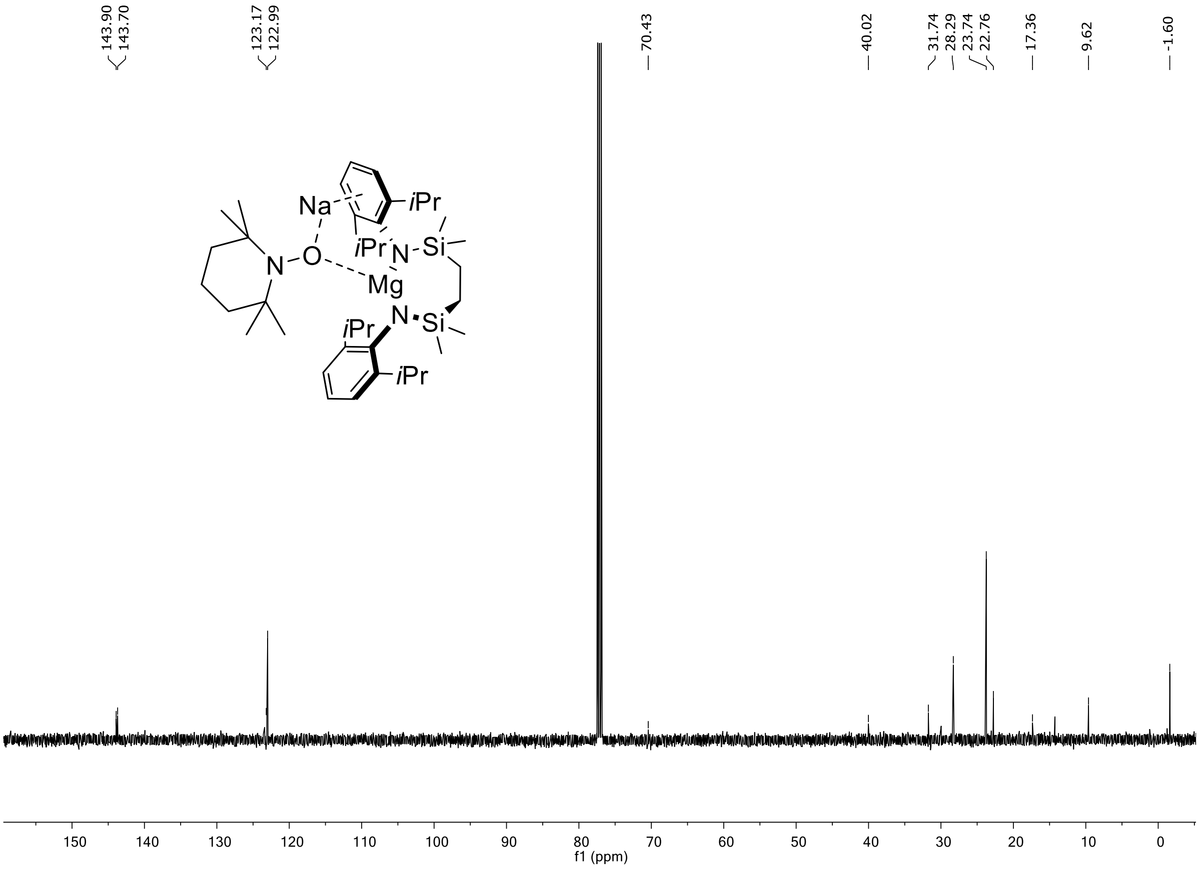

Figure S4: ^{1}H-^{13}C HSQC spectrum of compound **7**.

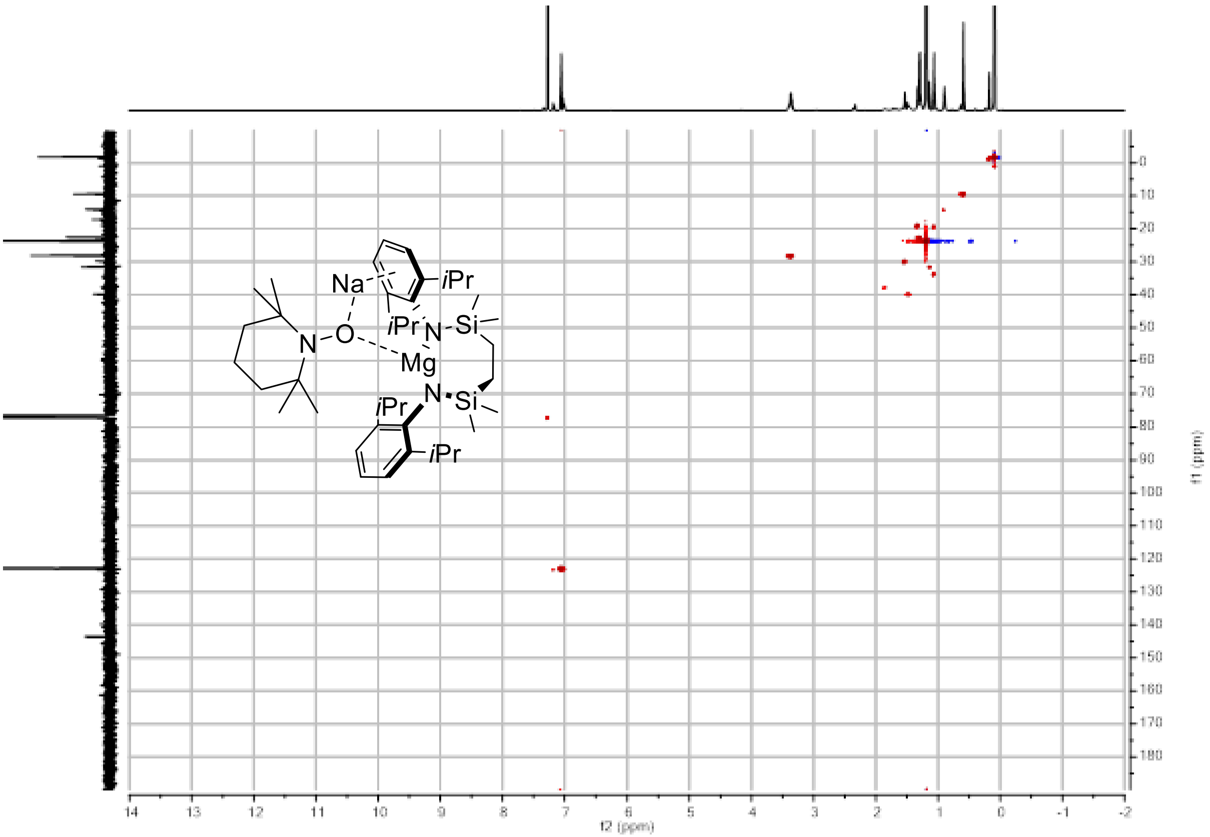

Figure S5: ^{1}H-^{13}C HMBC spectrum of compound **7**.

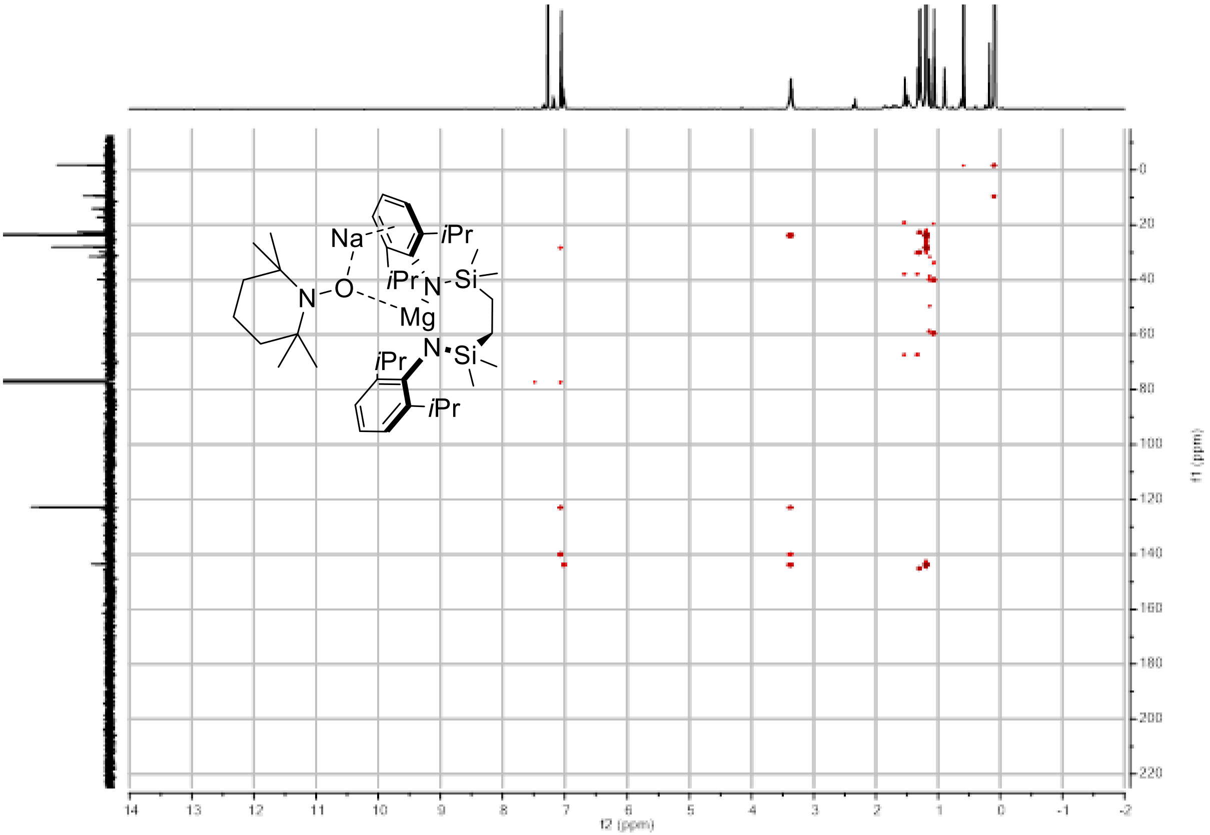

Figure S6: ^{1}H NMR (500 MHz, 298 K, Benzene-d_6) spectrum of **9**.

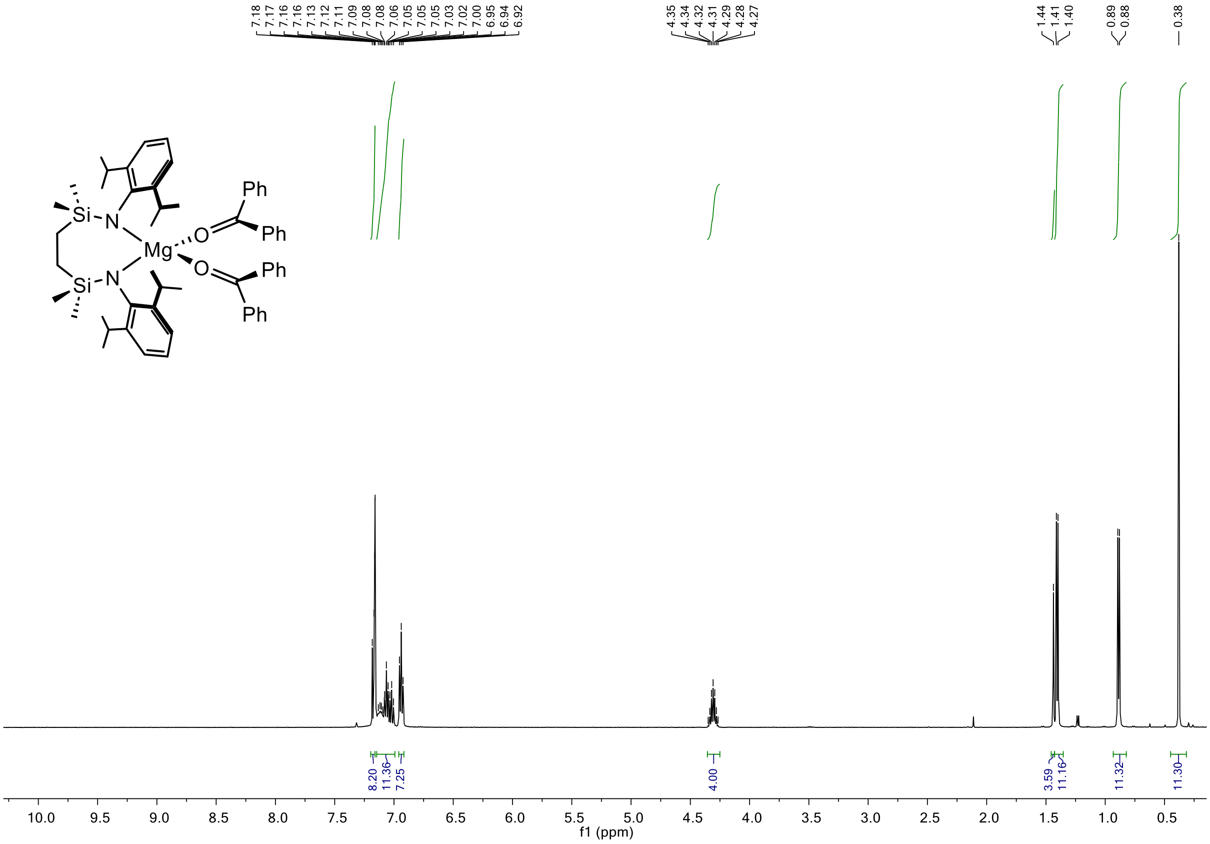

Figure S7: ^{13}C NMR (126 MHz, 298 K, Benzene-d_6) spectrum of **9**.

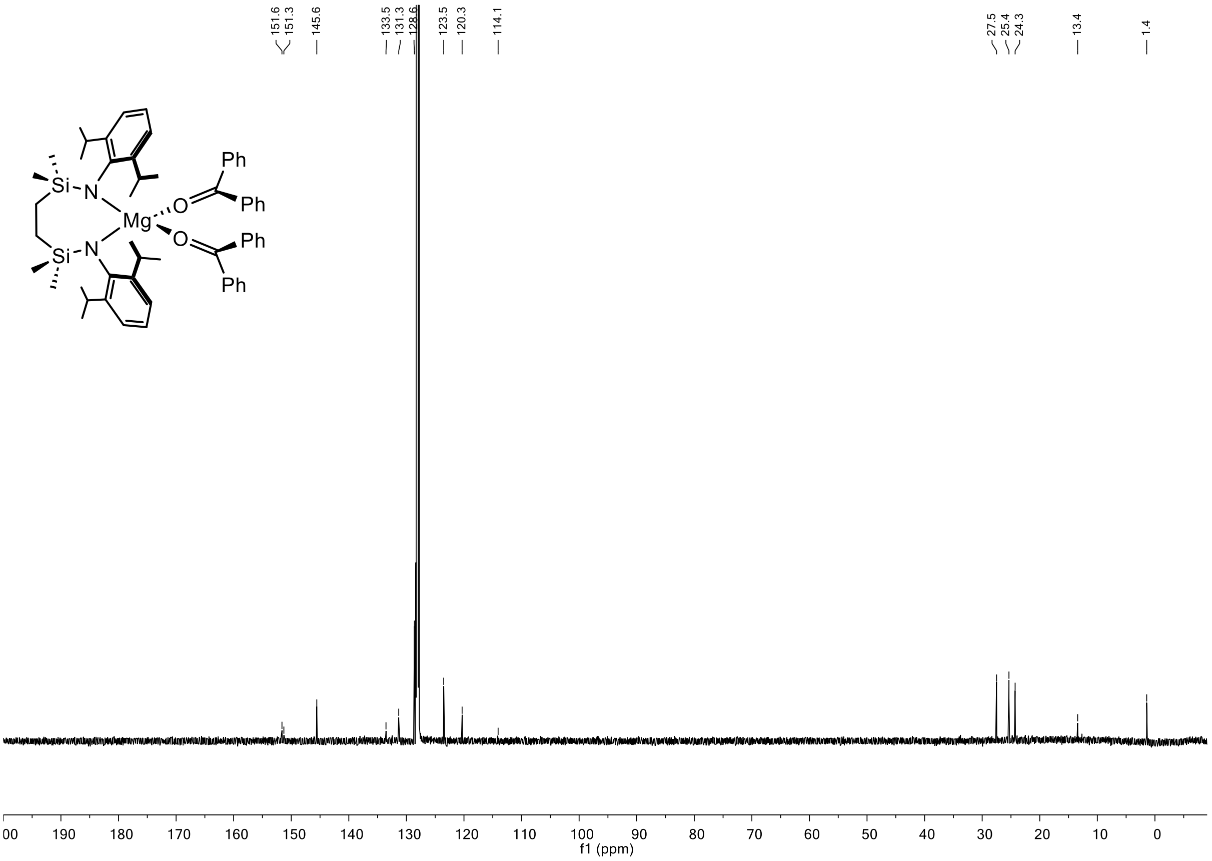

Figure S8: ^{1}H-^{1}H COSY NMR spectrum of **9**.

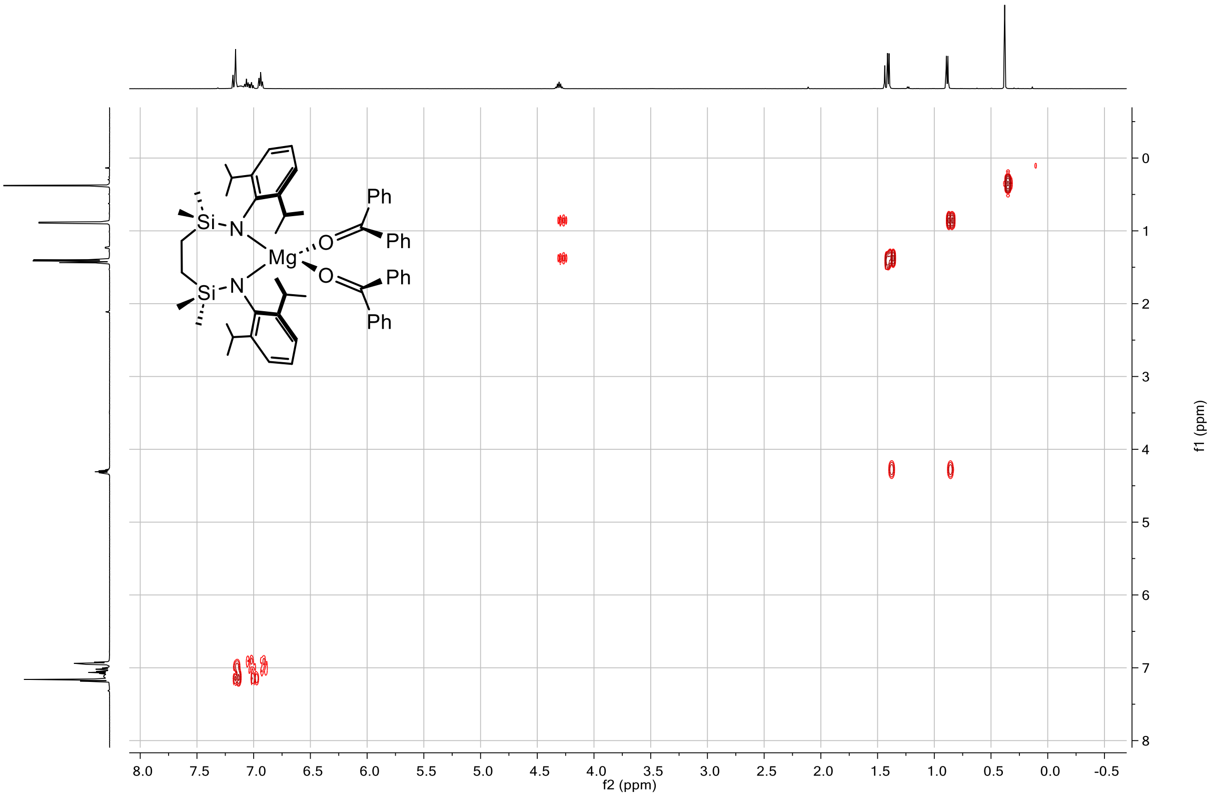

Figure S9: ¹H-¹³C HSQC NMR spectrum of **9**.

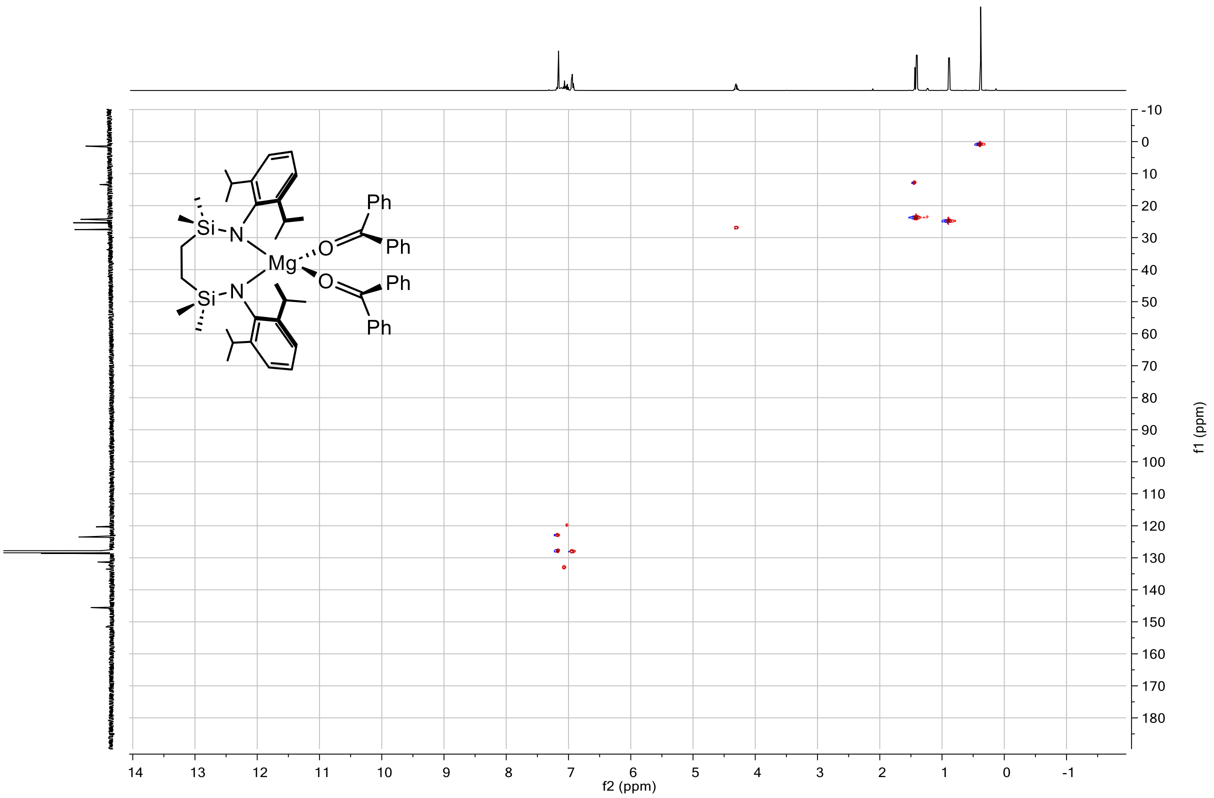

Figure S10: ¹H-¹³C HMBC NMR spectrum of **8**.

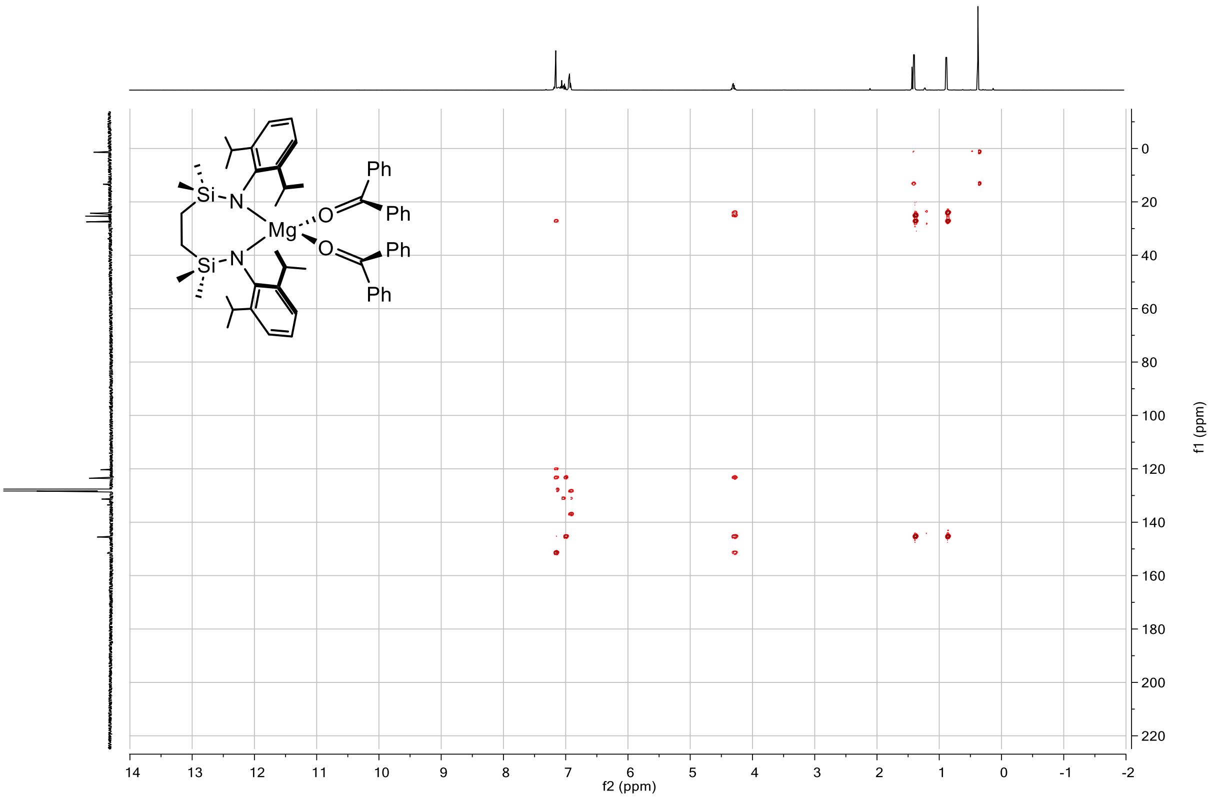

Figure S11: ^{1}H NMR spectrum (500 MHz, 298 K, Toluene-d_8) of **10**.

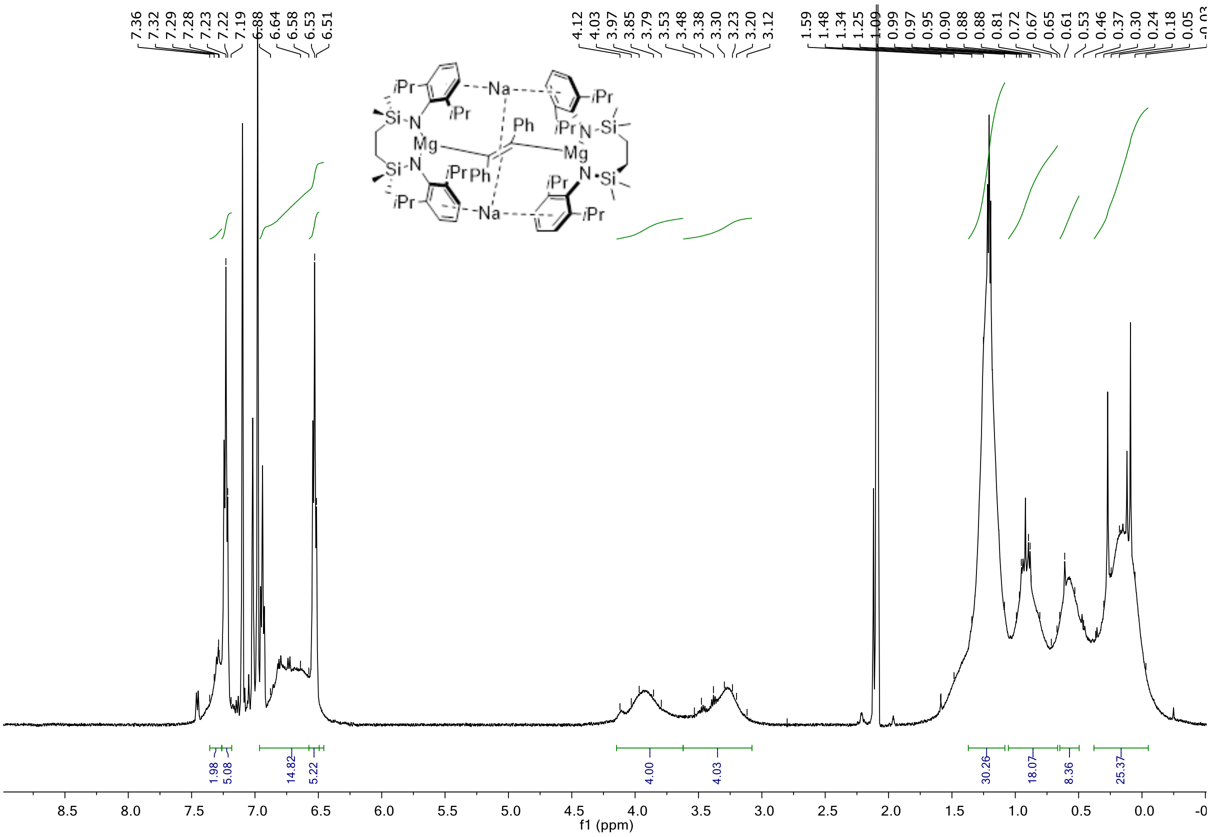

Figure S12: ^{13}C{^{1}H} NMR spectrum (101 MHz, 298 K, Toluene-d_8) of **10**.

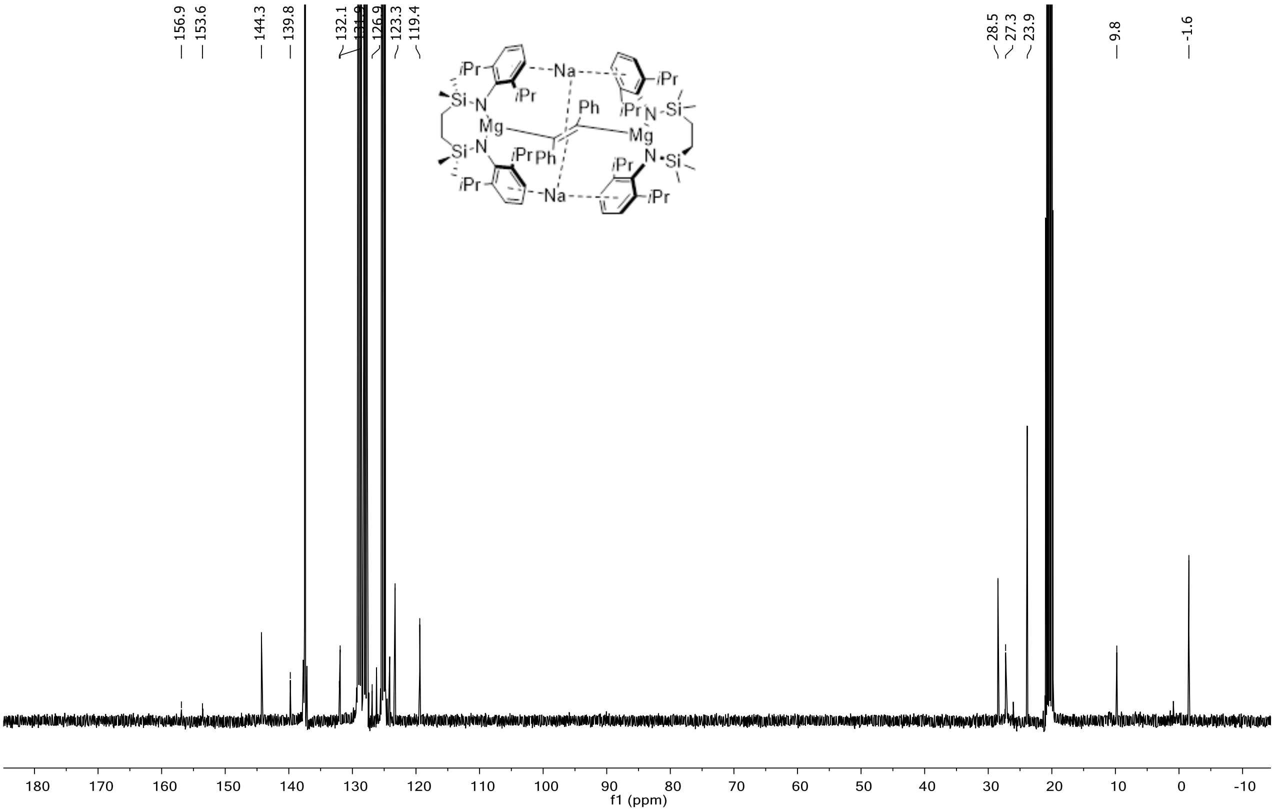

Figure S13: ^{1}H-^{13}C HSQC spectrum of **10**.

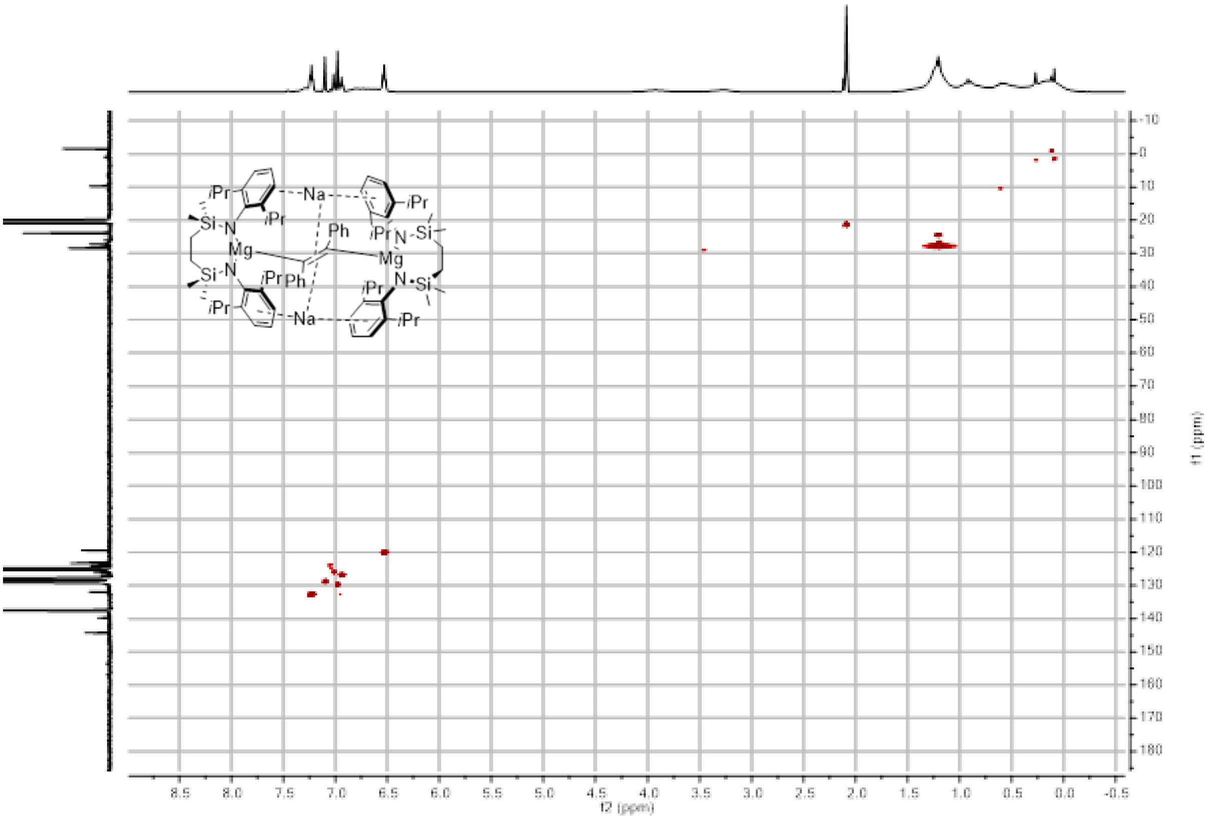

Figure S14: ^{1}H-^{13}C HMBC spectrum of **10**.

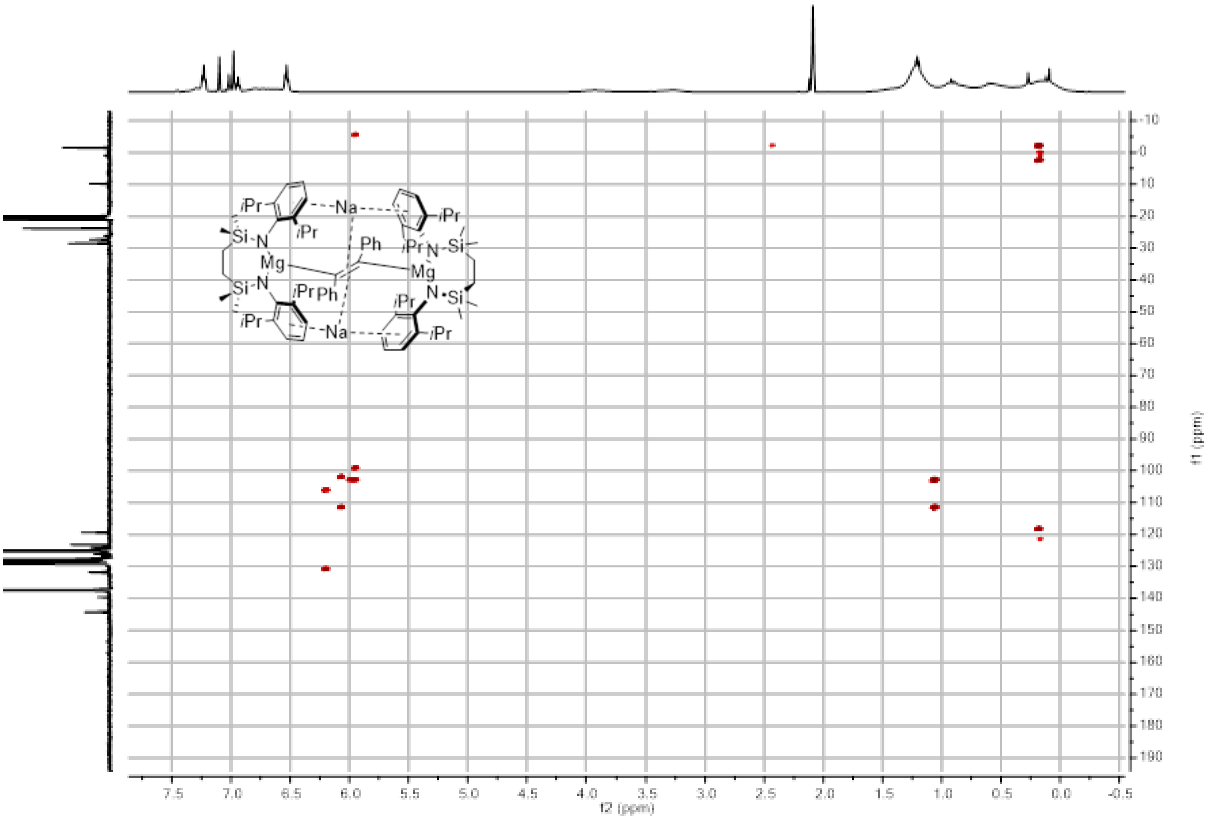

Crystallographic Details

Single Crystal X–ray diffraction data were collected on an Agilent SuperNova, diffractometer using either (**8** – **10**) CuKα (λ = 1.54184 Å) or (**6**) MoKα (λ = 0.71073 Å) radiation. In each case, the crystals were maintained at 150 K during data collection. Using Olex2,[2] the structures were solved with the olex2.solve[3] or ShelXT programs and refined using ShelXL[4].

The asymmetric unit comprises half of a dimer plus half of a benzene moiety in **6**. The remainder of the bimetallic complex is generated by a crystallographic 2-fold rotation axis, while the solvent is completed by virtue of inversion symmetry intrinsic to the space group. 80:20 disorder was taken into account for the benzene content with distance and planarity restraints being employed in the disordered region to assist convergence.

The hydrogen atoms attached to C61 and C62 were located and refined without restraints in the structure of **8**.

In **9**, the asymmetric unit equates to half of one molecule. The remainder is generated by the crystallographic, 2-fold rotation axis coincident with Mg1.

In addition to one molecule of the bimetallic dimer, the asymmetric unit plays host to a pair of toluene molecules in the structure of **10**. The bridging ligand in the dimer was treated for 58:42 disorder, while the toluene based on C85 was modelled to take account of 75:25 disorder. Distance and ADP restraints were employed, on merit, in disordered regions to assist convergence.

Table S1: Crystal Data and Structural Refinement for the Alkaline Earth Compounds **6** and **8** – **10**.

	**6**	**8**	**9**	**10**
Empirical formula	$C_{66}H_{106}Mg_2N_4Na_2OSi_4$	$C_{74}H_{110}Mg_2N_4Na_2Si_4$	$C_{56}H_{70}MgN_2O_2Si_2$	$C_{88}H_{126}Mg_2N_4Na_2Si_4$
Formula weight	1178.50	1262.61	883.63	1446.88
Crystal system	monoclinic	monoclinic	orthorhombic	monoclinic
Space group	*I*2/*a*	$P2_1/n$	*Pbcn*	$P2_1/c$
a/ Å	23.3563(4)	9.8837(1)	20.3152(5)	25.5518(5)
b/ Å	14.3910(2)	22.4079(1)	13.2445(3)	13.2671(2)
c/ Å	21.1181(3)	33.1579(2)	18.5748(5)	24.6303(4)
α/ °	90	90	90	90
β/ °	105.484(2)	93.601(1)	90	94.791(2)
γ/ °	90	90	90	90
U/ Å^3	6840.59(19)	7329.08(9)	4997.8(2)	8320.5(2)
Z	4	4	4	4
ρ_{calc}/ g cm^{-3}	1.144	1.144	1.174	1.155
μ/ mm^{-1}	0.160	1.353	1.087	1.253
F(000)	2560.0	2736.0	1904.0	3136.0
Crystal size/ mm^3	0.328 × 0.285 × 0.141	0.202 × 0.081 × 0.051	0.104 × 0.081 × 0.066	0.156 × 0.135 × 0.04
2Θ range for data collection/°	6.476 to 60.82	7.892 to 146.096	7.968 to 145.61	7.514 to 145.948
Index ranges	$-31 \le h \le 32$<br>$-20 \le k \le 20$<br>$-29 \le l \le 29$	$-12 \le h \le 9$<br>$-27 \le k \le 27$<br>$-41 \le l \le 40$	$-14 \le h \le 24$<br>$-16 \le k \le 10$<br>$-22 \le l \le 22$	$-30 \le h \le 31$<br>$-16 \le k \le 15$<br>$-23 \le l \le 30$
Reflections collected	56107	96648	12450	43508
Independent reflections, R_{int}	9474, 0.0239	14574, 0.0389	4880, 0.0361	16375, 0.0535
Data/restraints/parameters	9474/72/397	14574/0/799	4880/0/291	16375/610/1045
Goodness–of–fit on F^2	1.042	1.024	1.020	1.055
Final R_1, wR_2 [I>=2σ(I)]	0.0333, 0.0859	0.0380, 0.1021	0.0495, 0.1235	0.0742, 0.1870
Final R_1, wR_2 [all data]	0.0414, 0.0905	0.0429, 0.1064	0.0720, 0.1361	0.0994, 0.2022
Largest diff. peak/hole/ e Å^{-3}	0.32/-0.24	0.36/-0.32	0.30/-0.20	0.54/-0.46

Computational Details

DFT calculations were run with Gaussian 16 (C.01).[5] The Na, Mg, and Si centres were described with the Stuttgart RECPs and associated basis sets,[6] and the 6-31G** basis set was used for all other atoms (BS1).[7] A polarisation function was also added to Si (ζ_d = 0.284). Initial BP86 optimisations were performed using the 'grid = ultrafine' option,[8] with all stationary points being fully characterised via analytical frequency calculations as minima or transition states (all positive eigenvalues or one imaginary eigenvalue respectively). All energies were recomputed with a larger basis set featuring 6-311++G** basis sets on all atoms (BS2). Corrections for the effect of benzene (ε = 2.2706) solvent were run using the polarisable continuum model and BS1.[9] Single-point dispersion corrections to the BP86 results employed Grimme's D3 parameter set with Becke-Johnson damping as implemented in Gaussian.[10] Quantum Theory of Atoms in Molecules (QTAIM, AIMALL program[11]) analysis was performed on the BP86-optmised geometries $\mathbf{P_E}$, using the wavefunction file obtained with Gaussian 16 (C.01) at the BP86/BS2 level.

Breakdown of energy contributions

The following table details the evolution of the relative energies as the successive corrections to the initial SCF energy are included. Terms used are:

$\mathbf{\Delta E_{BSI}}$	SCF energy computed with the BP86 functional with BS1
$\mathbf{\Delta H_{BSI}}$	Enthalpy at 0 K with BS1
$\mathbf{\Delta G_{BSI}}$	Free energy at 298.15 K and 1 atm with BS1
$\mathbf{\Delta G_{BSI/C_6H_6}}$	Free energy corrected for C_6H_6 solvent with BS1
$\mathbf{\Delta G_{BSI/C_6H_6+D3BJ}}$	Free energy corrected for C_6H_6 and dispersion effects (D3BJ) with BS1
$\mathbf{\Delta E_{BS2}}$	SCF energy computed with the BP86 functional with BS2
$\mathbf{\Delta G_{C_6H_6}}$	Free energy corrected for BS2, D3BJ and C_6H_6 solvent

In each case the final data used in the main article are highlighted in bold.

Table S2: Relative energies (kcal mol^{-1}) for computed structures. Data in bold are those used in the main text. All energies are quoted relative to **I** at 0.0 kcal/mol.

	ΔE_{BSI}	ΔH_{BSI}	ΔG_{BSI}	$\Delta G_{BSI/C_6H_6}$	$\Delta G_{BSI/C_6H_6+D3BJ}$	ΔE_{BS2}	$\Delta G_{C_6H_6}$
**I**	0.0	0.0	0.0	0.0	0.0	0.0	**0.0**
**TS(I-II)**	20.6	79.7	15.3	34.2	49.7	18.7	**27.0**
**II**	17.9	77.5	8.9	38.4	60.1	16.7	**25.9**
**TS(II-III)**	19.5	78.4	10.6	38.3	58.4	18.1	**25.7**
**III**	16.4	76.1	6.3	33.8	54.6	13.0	**19.0**
**TS(III-IV)**	43.2	103.2	52.6	21.5	-0.1	43.1	**30.8**
**IV**	6.9	67.4	17.8	-16.1	-41.0	8.8	**-5.1**
**TS(IV-V)**	29.7	89.4	39.4	5.5	-19.0	30.1	**13.9**
**V**	21.5	81.6	29.7	4.7	-12.7	22.1	**12.1**
**TS(V-VI)**	28.0	87.2	33.0	13.8	0.8	26.8	**17.1**
**VI**	15.1	75.3	19.1	10.9	5.8	16.0	**13.0**
**TS(VI-VII)**	19.3	78.9	27.4	8.5	-4.0	21.1	**17.0**
**VII**	-10.7	49.5	-2.5	-25.5	-40.4	-10.6	**-17.5**
**TS(VII-VIII)**	-1.3	58.2	8.9	-23.0	-46.0	0.0	**-11.6**
**VIII**	-5.3	54.8	5.9	-31.7	-60.5	-2.9	**-18.2**
**VIII**	-5.3	54.8	5.9	-31.7	-60.5	-2.9	**-18.2**
**TS(VIII-IX)**	-4.0	56.0	10.2	-36.3	-71.9	-0.7	**-18.9**
**INT(PE)**	-25.9	35.8	-9.7	-66.3	-112.1	-20.4	**-44.1**
**P_Z**	-4.0	57.9	14.5	-51.3	-103.4	0.7	**-27.9**
**P_E**	-32.1	29.5	-13.0	-82.3	-138.4	-26.2	**-57.4**

QTAIM results

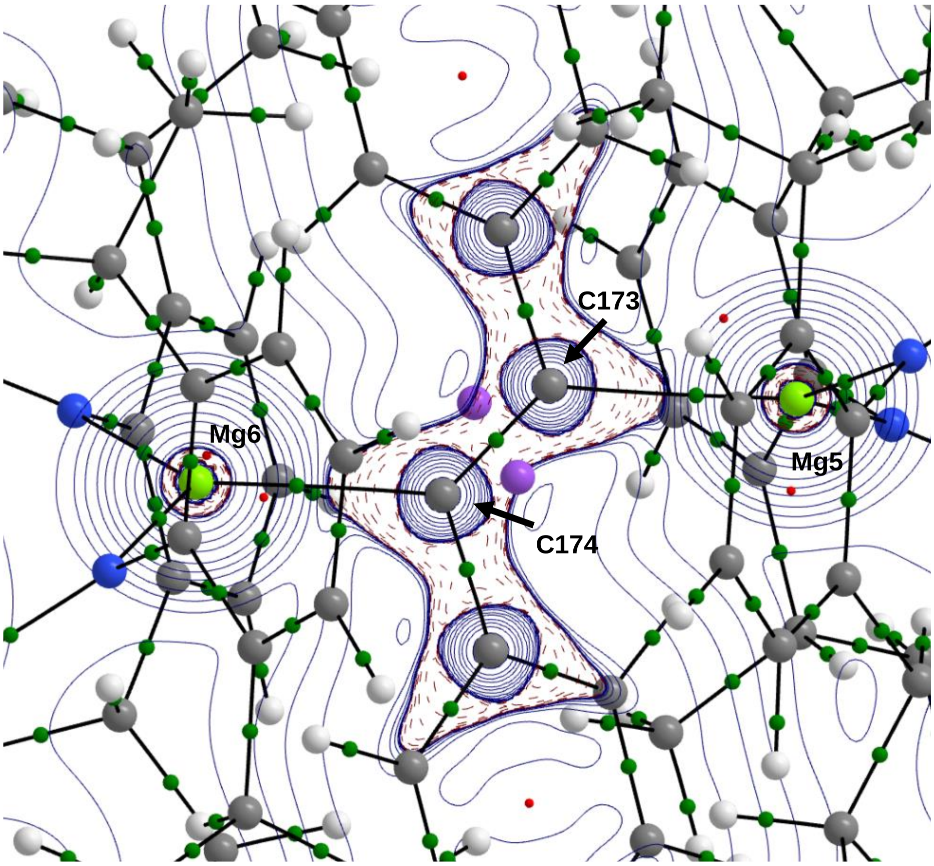

Figure S15. Contour plot of $\mathbf{P_E}$

Table S3. Selected BCP data for $\mathbf{9^{Na}}$ (BCPs under the threshold of 0.005 are denoted with an asterisk).

BCP	ρ(r)	∇²ρ(r)	ε	G(r)	V(r)	H(r)
Mg5 - C173	0.041080	+0.164981	0.038062	+0.041893	-0.042541	–0.000648
Mg6 - C174	0.041075	+0.164944	0.038099	+0.041884	-0.042533	–0.000648
C173 - C174	0.299634	-0.749279	0.152185	+0.113956	-0.415231	–0.301275

Table S4. Selected QTAIM atomic data for $\mathbf{9^{Na}}$.

Atom	q(A)	L(r)	N(r)	Vol(r)	Loc(r)
Mg6	+1.635993	-0.000164	10.364007	50.630796	96.090640
Mg5	+1.636393	+0.000068	10.363607	50.535872	96.094003
C174	-0.620776	-0.000179	6.620776	113.926706	70.962207
C173	-0.620428	+0.000004	6.620428	113.853992	70.963385

References

1. Liu, H. Y.; Schwamm, R. J.; Neale, S. E.; Hill, M. S.; McMullin, C. L.; Mahon, M. F., *J. Am. Chem. Soc.* **2021,** *143* (42), 17851-17856.

2. Dolomanov, O. V.; Bourhis, L. J.; Gildea, R. J.; Howard, J. A. K.; Puschmann, H., *J. Appl. Cryst.* **2009**, *42*, 339-341.

3. Sheldrick, G. M., *Acta Cryst*. **2015**, *A71*, 3-8.

4. Sheldrick, G. M., *Acta Cryst*. **2015**, *C71*, 3-8.

5. M. J. Frisch, G. W. Trucks, H. B. Schlegel, G. E. Scuseria, M. A. Robb, J. R. Cheeseman, G. Scalmani, V. Barone, G. A. Petersson, H. Nakatsuji, X. Li, M. Caricato, A. V. Marenich, J. Bloino, B. G. Janesko, R. Gomperts, B. Mennucci, H. P. Hratchian, J. V. Ortiz, A. F. Izmaylov, J. L. Sonnenberg, Williams, F. Ding, F. Lipparini, F. Egidi, J. Goings, B. Peng, A. Petrone, T. Henderson, D. Ranasinghe, V. G. Zakrzewski, J. Gao, N. Rega, G. Zheng, W. Liang, M. Hada, M. Ehara, K. Toyota, R. Fukuda, J. Hasegawa, M. Ishida, T. Nakajima, Y. Honda, O. Kitao, H. Nakai, T. Vreven, K. Throssell, J. A. Montgomery Jr., J. E. Peralta, F. Ogliaro, M. J. Bearpark, J. J. Heyd, E. N. Brothers, K. N. Kudin, V. N. Staroverov, T. A. Keith, R. Kobayashi, J. Normand, K. Raghavachari, A. P. Rendell, J. C. Burant, S. S. Iyengar, J. Tomasi, M. Cossi, J. M. Millam, M. Klene, C. Adamo, R. Cammi, J. W. Ochterski, R. L. Martin, K. Morokuma, O. Farkas, J. B. Foresman, D. J. Fox, Wallingford, CT, 2016.

6. D. Andrae, U. Häußermann, M. Dolg, H. Stoll, H. Preuß, *Theor. Chim. Acta* 1990, 77, 123-141.

7. (a) P. C. Hariharan, J. A. Pople, *Theor. Chim. Acta* 1973, 28, 213-222; (b) W. J. Hehre, R. Ditchfield, J. A. Pople, *J. Chem. Phys.* 1972, 56, 2257-2261.

8. (a) A. D. Becke, *Phys. Rev. A* 1988, 38, 3098-3100; (b) J. P. Perdew, *Phys. Rev. B* 1986, 33, 8822-8824.

9. J. Tomasi, B. Mennucci, R. Cammi, *Chem. Rev.* 2005, 105, 2999-3094.

10. S. Grimme, S. Ehrlich, L. Goerigk, *J. Comp. Chem.* 2011, 32, 1456-1465.

11. AIMAll (Version 19.10.12), T. A. Keith, TK Gristmill Software, Overland Park KS, USA, 2019 (aim.tkgristmill.com).

Computed Energies (in Hartrees) for Calculated Structures

PhCCPh
SCF (BP86) Energy = -539.448544253
Enthalpy 0K = -539.262694
Enthalpy 298K = -539.250278
Free Energy 298K = -539.300969
Lowest Frequency = 22.9750 cm^{-1}
Second Frequency = 44.1588 cm^{-1}
SCF (BP86-D3BJ) Energy = -539.491978901
SCF (C6H6) Energy = -539.450735610
SCF (BS2) Energy = -539.580362543

TS(I-II)
SCF (BP86) Energy = -2583.67752820
Enthalpy 0K = -2582.249984
Enthalpy 298K = -2582.152757
Free Energy 298K = -2582.384425
Lowest Frequency = -15.6126 cm^{-1}
Second Frequency = 12.5003 cm^{-1}
SCF (BP86-D3BJ) Energy = -2584.15709441
SCF (C6H6) Energy = -2583.70000906
SCF (BS2) Energy = -4449.47407282

II
SCF (BP86) Energy = -2583.68183314
Enthalpy 0K = -2582.254479
Enthalpy 298K = -2582.156325
Free Energy 298K = -2582.394698
Lowest Frequency = 6.4776 cm^{-1}
Second Frequency = 10.6878 cm^{-1}
SCF (BP86-D3BJ) Energy = -2584.15145137
SCF (C6H6) Energy = -2583.70326405
SCF (BS2) Energy = -4449.48086632

III
SCF (BP86) Energy = -2583.68428408
Enthalpy 0K = -2582.257046
Enthalpy 298K = -2582.158464
Free Energy 298K = -2582.398871
Lowest Frequency = 9.4319 cm^{-1}
Second Frequency = 10.7907 cm^{-1}
SCF (BP86-D3BJ) Energy = -2584.15537967
SCF (C6H6) Energy = -2583.70915455
SCF (BS2) Energy = -4449.48516456

TS(II-III)
SCF (BP86) Energy = -2583.67939734
Enthalpy 0K = -2582.252376
Enthalpy 298K = -2582.154895
Free Energy 298K = -2582.391891
Lowest Frequency = -47.5194 cm^{-1}
Second Frequency = 8.9163 cm^{-1}
SCF (BP86-D3BJ) Energy = -2584.15153303
SCF (C6H6) Energy = -2583.70090236
SCF (BS2) Energy = -4449.47899403

TS(III-IV)
SCF (BP86) Energy = -3123.09005195
Enthalpy 0K = -3121.475404
Enthalpy 298K = -3121.365560
Free Energy 298K = -3121.626026
Lowest Frequency = -92.1870 cm^{-1}
Second Frequency = 11.3072 cm^{-1}
SCF (BP86-D3BJ) Energy = -3123.67218514
SCF (C6H6) Energy = -3123.11182687
SCF (BS2) Energy = -4989.01546540

IV
SCF (BP86) Energy = -3123.14789643
Enthalpy 0K = -3121.532051
Enthalpy 298K = -3121.422689
Free Energy 298K = -3121.681523
Lowest Frequency = 8.8036 cm^{-1}
Second Frequency = 15.3057 cm^{-1}
SCF (BP86-D3BJ) Energy = -3123.73529005
SCF (C6H6) Energy = -3123.16645311
SCF (BS2) Energy = -4989.07302219

TS(IV-V)
SCF (BP86) Energy = -3123.11157858
Enthalpy 0K = -3121.496480
Enthalpy 298K = -3121.387582
Free Energy 298K = -3121.646997
Lowest Frequency = -34.5402 cm^{-1}
Second Frequency = 7.5200 cm^{-1}
SCF (BP86-D3BJ) Energy = -3123.69836612
SCF (C6H6) Energy = -3123.13261819
SCF (BS2) Energy = -4989.03902527

V
SCF (BP86) Energy = -3123.12468193
Enthalpy 0K = -3121.510200
Enthalpy 298K = -3121.400078
Free Energy 298K = -3121.662415
Lowest Frequency = 10.3684 cm^{-1}
Second Frequency = 13.3861 cm^{-1}
SCF (BP86-D3BJ) Energy = -3123.70002153
SCF (C6H6) Energy = -3123.14538183
SCF (BS2) Energy = -4989.05141818

TS(V-VI)
SCF (BP86) Energy = -3123.11428986
Enthalpy 0K = -3121.501455
Enthalpy 298K = -3121.391145
Free Energy 298K = -3121.657194
Lowest Frequency = -69.4405 cm^{-1}
Second Frequency = 6.1680 cm^{-1}
SCF (BP86-D3BJ) Energy = -3123.68264445
SCF (C6H6) Energy = -3123.13791649
SCF (BS2) Energy = -4989.04239922

VI
SCF (BP86) Energy = -3123.13491242
Enthalpy 0K = -3121.520913
Enthalpy 298K = -3121.410064
Free Energy 298K = -3121.679425
Lowest Frequency = 5.3474 cm^{-1}
Second Frequency = 8.3760 cm^{-1}
SCF (BP86-D3BJ) Energy = -3123.69068073
SCF (C6H6) Energy = -3123.15512263
SCF (BS2) Energy = -4989.06323575

TS(VI-VII)
SCF (BP86) Energy = -3123.12812995
Enthalpy 0K = -3121.513856
Enthalpy 298K = -3121.404418
Free Energy 298K = -3121.666147
Lowest Frequency = -46.0189 cm^{-1}
Second Frequency = 5.1311 cm^{-1}
SCF (BP86-D3BJ) Energy = -3123.69577776
SCF (C6H6) Energy = -3123.14699122
SCF (BS2) Energy = -4989.05287644

VII
SCF (BP86) Energy = -3123.17602055

Enthalpy 0K = -3121.560989
Enthalpy 298K = -3121.451147
Free Energy 298K = -3121.713784
Lowest Frequency = 5.5722 cm^{-1}
Second Frequency = 8.6559 cm^{-1}
SCF (BP86-D3BJ) Energy = -3123.74740976
SCF (C6H6) Energy = -3123.19749401
SCF (BS2) Energy = -4989.10175446

TS(VII-VIII)
SCF (BP86) Energy = -3123.16109342
Enthalpy 0K = -3121.546448
Enthalpy 298K = -3121.437293
Free Energy 298K = -3121.695645
Lowest Frequency = -67.0562 cm^{-1}
Second Frequency = 6.5923 cm^{-1}
SCF (BP86-D3BJ) Energy = -3123.74543329
SCF (C6H6) Energy = -3123.18051567
SCF (BS2) Energy = -4989.08464500

VIII
SCF (BP86) Energy = -3123.16738417
Enthalpy 0K = -3121.552374
Enthalpy 298K = -3121.442684
Free Energy 298K = -3121.700403
Lowest Frequency = 11.5345 cm^{-1}
Second Frequency = 16.0657 cm^{-1}
SCF (BP86-D3BJ) Energy = -3123.76098442
SCF (C6H6) Energy = -3123.18511273
SCF (BS2) Energy = -4989.08908678

TS(VIII-P)
SCF (BP86) Energy = -3123.16534881
Enthalpy 0K = -3121.549353
Enthalpy 298K = -3121.440915
Free Energy 298K = -3121.693535
Lowest Frequency = -43.6002 cm^{-1}
Second Frequency = 13.7060 cm^{-1}
SCF (BP86-D3BJ) Energy = -3123.76979570
SCF (C6H6) Energy = -3123.18164535
SCF (BS2) Energy = -4989.08570533

INT(P_E)
SCF (BP86) Energy = -3123.20014075
Enthalpy 0K = -3121.581335
Enthalpy 298K = -3121.472981
Free Energy 298K = -3121.725257
Lowest Frequency = 16.0552 cm^{-1}
Second Frequency = 17.7476 cm^{-1}
SCF (BP86-D3BJ) Energy = -3123.82091681
SCF (C6H6) Energy = -3123.21304314
SCF (BS2) Energy = -4989.11589496

P_Z
SCF (BP86) Energy = -3123.16527808
Enthalpy 0K = -3121.545398
Enthalpy 298K = -3121.437759
Free Energy 298K = -3121.686707
Lowest Frequency = 18.3300 cm^{-1}
Second Frequency = 22.0513 cm^{-1}
SCF (BP86-D3BJ) Energy = -3123.79584455
SCF (C6H6) Energy = -3123.17948093
SCF (BS2) Energy = -4989.08278195

P_E
SCF (BP86) Energy = -3123.21014154
Enthalpy 0K = -3121.590763
Enthalpy 298K = -3121.483064

Free Energy 298K = -3121.730473
Lowest Frequency = 16.7361 cm^{-1}
Second Frequency = 21.7168 cm^{-1}
SCF (BP86-D3BJ) Energy = -3123.84723087
SCF (C6H6) Energy = -3123.22228128
SCF (BS2) Energy = -4989.12637055
